# Capturing Heterogeneous Time‐Variation in Covariate Effects in Non‐Proportional Hazard Regression Models

**DOI:** 10.1002/bimj.70179

**Published:** 2026-09-20

**Authors:** Niklas Hagemann, Thomas Kneib, Kathrin Möllenhoff

**Affiliations:** ^1^ Department of Biostatistics Harvard T.H. Chan School of Public Health Harvard University Boston USA; ^2^ Institute of Medical Statistics and Computational Biology Faculty of Medicine University of Cologne Göttingen Germany; ^3^ Chair of Statistics and Campus Institute Data Science Georg August University Göttingen Cologne Germany

**Keywords:** effect heterogeneity, factor smooth interactions, functional random coefficients, functional random effects, hazard regression, non‐proportional hazards, piecewise exponential model, penalized splines, random wiggly curves, time‐varying effects

## Abstract

A central focus in survival analysis is examining how covariates affect survival time. These covariate effects are often found to be either time‐varying, heterogeneous—such as being specific to patients, treatments, or subgroups—or exhibit both characteristics simultaneously. While the standard model, the Cox proportional hazards model, allows neither time‐varying nor heterogeneous effects, several extensions to the Cox model as well as alternative modeling frameworks have been introduced. However, no unified framework for incorporating heterogeneously time‐varying effects of covariates has been proposed. Such effects occur when a covariate affects survival not only in a heterogeneous and time‐varying manner, but when the time‐variation is also heterogeneous. We propose to model such effects by introducing heterogeneously time‐varying coefficients to piecewise exponential additive mixed models. We deploy functional random effects, also known as factor smooths, to model such coefficients as the interaction effect of heterogeneity and time‐variation. Our approach allows for non‐linear time‐effects due to being based on penalized splines and uses an efficient random effects basis to model the heterogeneity. Using a penalized basis prevents overfitting in case of absence of such effects. In addition, the penalization mostly solves the problem of choosing the number of intervals which is usually present in unregularized piecewise exponential approaches. We demonstrate the superiority of our approach in comparison to competitors by means of a simulation study. Finally, the practical application and relevance are outlined by presenting a brain tumor case study.

## 1 Introduction

One of the major topics in survival analysis is analyzing the effect covariates have on the survival time. Frequently, the effects of these covariates can be observed to be either time‐varying, heterogeneous, that is, patient‐, treatment‐, or subgroup‐specific, or even both. If the goal is to analyze the effects of covariates on the survival time, hazard regression models play a critical role. They estimate the hazard function, which represents the instantaneous rate of occurrence of the event at a given time, conditional on survival up to that time. By incorporating covariates, hazard regression models allow to assess the impact of various factors on the hazard rate and, hence, to identify significant effects. The widely used standard model is the *Cox proportional hazards model* (Cox 1972) where the hazard rate of an observation i∈{1,…,n} with corresponding covariate vector $x_{i}$ is given by

$$\lambda_{i}\left(t|x_{i}\right)=\lambda_{0}\left(t\right)\exp\left(x_{i}^{⊤}\beta \right),$$

where β is the vector of regression coefficients. The assumption of proportionality of the hazards results from the model being strictly split into the time‐dependent baseline hazard $\lambda_{0}\left(t\right)$ and the time‐constant covariate effects $\exp(x_{i}^{⊤}\beta )$. In addition, the Cox model assumes the covariate effects to be linear on the log‐hazard scale.

These strict assumptions are often not fulfilled in practice (Jachno et al. 2019; Li et al. 2015). Frequently, this is caused by non‐proportional hazards, that is, the covariate effects are time‐dependent. In addition, in many cases the effects might not be linear but of a more complex form. In particular, heterogeneity in terms of treatment‐specific, subgroup‐specific (e.g., gender‐specific) or individual effects might be present which cannot be captured properly by linear effects. This raised criticism of the use of methods that rely on these assumptions, particularly in causal inference. For example, Dumas and Stensrud (2025) describe how incorrectly assuming proportional hazards can invalidate causal inference methods.

In the following, we will give an overview of extensions to the Cox model for both non‐proportional hazards and non‐linear effects. Subsequently, we will proceed to approaches that do not add specific extensions to the Cox model but introduce flexible hazard regression frameworks, focusing on piecewise exponential approaches. Based on this framework, we will discuss the practical demand for heterogeneously time‐varying effects and introduce the underlying concept of functional random coefficients.

On the one hand, flexible extensions of the Cox model were introduced to relax the proportional hazards assumption: The most commonly used may be the stratified Cox model in which for each level of a categorical variable a separate baseline hazard is fitted. Alternatively, several studies (see, e.g., Murphy and Sen 1991; Zucker and Karr 1990) propose to include time‐varying coefficients in order to capture time‐dependent covariate effects. In addition, Andersen and Gill (1982) introduced an approach to include time‐dependent covariates and Cai et al. (2007) discuss interaction effects of time‐dependent and time‐constant covariates.

On the other hand, extensions of the Cox model have been introduced to allow for more complex effects: Gray (1992) added non‐linear smooth spline‐based covariate effects and Hess (1994) uses such effects to express covariate effects as a function of time. In addition, Kooperberg et al. (1995), Stone et al. (1997, section 7) and Gu (1996) discuss the construction of time‐varying effects as non‐linear interactions of covariates and time. Regarding the heterogeneity, again the stratified Cox model can be mentioned, where the baseline hazard can be group‐specific. However, a more natural way to account for heterogeneous effects, which also allows for more general types of heterogeneity, is to introduce random effects leading to frailty models (Ripatti and Palmgren 2000; Therneau et al. 2003; Vaupel et al. 1979).

More recently, based on the work of Andersen and Gill (1982) and Therneau and Grambsch (2000), extending the Cox model by non‐linear spline‐based time‐varying effects has been proposed (see, e.g., Section 4.2 of Therneau et al. 2024). While this approach also allows for linearly interacting continuous effects, its capability for interactions of other types of effects, such as categorical variables, and non‐linear interactions, remains underexplored.

In contrast to adding specific extensions to the Cox model, several recent studies have aimed to introduce a new flexible hazard regression framework: Kneib and Fahrmeir (2007) introduce *Cox‐type structured hazard regression models*

(1) $$\begin{array}{c} \lambda_{i}\left(t|x_{i}\right)=\lambda_{0}\left(t\right)\exp\left(\;\sum_{k=1}^{K}f_{k}\left(x_{i},t\right)\right)=\exp\left({\tilde{\lambda }}_{0}\left(t\right)+\;\sum_{k=1}^{K}f_{k}\left(x_{i},t\right)\right), \end{array}$$

where ${\tilde{\lambda }}_{0}\left(t\right)$ is the log‐baseline hazard and $f_{k},k=1,\text{…},K$, denotes the K covariate effects with $f_{k}\left(x_{i},t\right)$ being shorthand for $f_{k}\left(x_{i1},\text{…},x_{ip},t\right)$, p being the number of covariates, and any given $f_{k}$ will often use only one or two of the covariates. The covariate effects can represent different types of effects, for example, linear effects, smooth (spline‐based) effects, time‐varying effects or random effects/frailty. The inclusion of time‐varying effects allows for explicit modeling of non‐proportional hazards. In contrast to the aforementioned most recent extensions to the Cox model, these approaches explicitly include non‐linear interactions, such as tensor product interaction, providing a sufficient basis to introduce interactions of time effects and heterogeneity. This approach was further investigated by Hofner et al. (2013, 2011). The corresponding inference is conducted by mixed model‐based penalized likelihood estimation. However, the log‐likelihood involves an integral over the hazard rate. Hence, the optimization problem lacks a closed form and the estimation relies on numerical integration. Alternatively, Hennerfeind et al. (2006) proposed a Bayesian estimation scheme for such models. However, this approach is based on the same log‐likelihood and, therefore, also lacks a closed form.

Another approach to introduce models of the form (1) is given by *piecewise exponential additive mixed models* (PAMM; Bender et al. 2018; Bender and Scheipl 2018) which generalizes the concept of *piecewise exponential models* (PEM; Friedman 1982) from linear to additive predictor terms. The underlying idea is to divide the time axis into a finite number of intervals and assume the hazard rate to be piecewise constant within these intervals. While manually choosing the interval cut points is a challenging task and a frequent source of criticism for PEMs, PAMMs avoid the arbitrary choice of cut points by using a penalized regression spline rather than a step function as baseline hazard. Due to penalization preventing overfitting, the number of cut points can then just be set to maximal flexibility. Under the assumption of piecewise constant hazard rates, restructuring the data leads to the likelihood of the survival model being proportional to the one of a Poisson regression model (see Section 2 of Bender et al. (2018) for details). Hence, both models are equivalent with respect to their maximum likelihood estimators and the model parameters are estimated based on the Poisson model. Therefore, the estimation can be based on the closed form of the Poisson likelihood. This enables the utilization of existing efficient methods and implementations for generalized linear models (GLMs) in the case of PEMs and generalized additive (mixed) models (GAMs/GAMMs) in the case of PAMMs.

**FIGURE 1:**
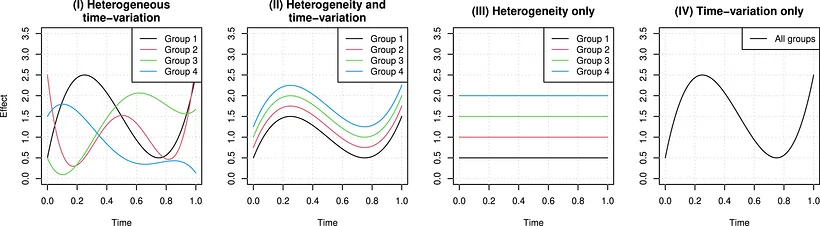
Heterogeneous time‐variation (first subplot) compared to the combination of heterogeneity and time‐variation (second subplot), heterogeneity only (third subplot), time variation only (fourth subplot). The shown effects correspond to the effect of $x_{2}$ in the DGPs in the four scenarios (I–IV) of the simulation study in Section 4.

While these approaches propose several flexible effects such as non‐linear, time‐varying, and random effects, as well as bivariate interaction effects, none of them include heterogeneously time‐varying effects of covariates. Such effects occur if a covariate affects the survival time not only in a heterogeneous and time‐varying manner, but the time‐varying effect is heterogeneous, too. This is exemplarily shown in Figure 1, where the first subplot displays heterogeneously time‐varying effects. In contrast, the second subplot shows an effect that is heterogeneous and time‐varying, but the time‐varying effect is not heterogeneous itself. The third and fourth subplots display the corresponding main effects. A typical example would be that the effect of a covariate is treatment‐specific, time‐varying and that its time‐variation is also treatment‐specific, for example, decreasing for an intervention but increasing for a placebo. To the best of our knowledge, this study is the first to propose heterogeneously time‐varying covariate effects in hazard regression models. Wong et al. (2011) address a similar problem, but their approach is only applicable to discrete survival data. As a result, it is also limited with regard to the types of time effects that can be captured, as it does not allow for continuous time effects.

Based on the framework of PAMMs, we introduce a heterogeneously time‐varying coefficient of a covariate effect k∈{1,…,K} within model (1) as

$$f_{kg}\left(t\right)\cdot x_{ik},$$

where g∈{1,…,G} is the grouping variable. Unlike approaches that focus on cluster/subgroup detection (e.g., Bertolet et al. 2016; Wang et al. 2022), this approach aims to incorporate a deterministic explanatory grouping variable such as treatment groups. Besides treatments, the grouping can also correspond to characteristics of the participants (e.g., gender or subdiagnoses), characteristics of the study (e.g., centers in multicenter studies) or even individual heterogeneity, that is, G=n. We propose to model such heterogeneously time‐varying coefficients based on *functional random effects* (FRE; Kneib et al. 2019). FREs can be seen as a non‐linear generalization of random intercepts and slopes, allowing to model whole non‐linear time curves group‐specifically. This leads to *functional random coefficients*, which Hagemann et al. (2024) recently proposed to use to capture heterogeneous time‐variation in covariate effects. Their study focuses on conditional logit models, a class of models commonly used in marketing research, but the approach is directly generalizable to other GAMs and can therefore be applied to PAMMs as well. Functional random effects are also known as *factor smooth interactions* or *random wiggly curves* (Wood 2017) and are essentially tensor product interactions of smooth effects and random effects.

This paper is structured as follows: In Section 2, piecewise exponential hazard regression models including the corresponding inference are succinctly discussed. In Section 3, we introduce subgroup‐specific time variation in covariate effects using functional random coefficients. A simulation study is conducted in Section 4 to show the ability of our approach to capture these effects as proposed. In addition, the simulations demonstrate that the penalized approach prevents overfitting in the absence of such effects. Section 5 illustrates the method and outlines its practical relevance by investigating the effect of fraction genome altered as a predictor of survival time in patients with brain tumors. Finally, Section 6 closes with a discussion.

## 2 Piecewise Exponential Hazard Regression Models

### 2.1 Piecewise Exponential Models

Piecewise exponential models (PEM; Friedman 1982) are an alternative to classical approaches in survival regression, especially to the Cox model. Their main advantage is that the corresponding inference can be based on a Poisson model and, hence, can make use of existing tools for generalized linear models (GLMs) and generalized additive models (GAMs). Consequently, within this framework, one can model time effects just as any other covariate effect and easily include them in interaction effects. They require the partition of the time axis into a finite number of intervals and assume the hazard rate to be constant within each interval. The piecewise exponential model is defined as

(2) $$\lambda_{i}\left(t|x_{i}\right)=\lambda_{0}\left(t_{j}\right)\exp\left(\eta \left(x_{i},t_{j}\right)\right)\;\forall \;t\in \left(\kappa_{j-1},\kappa_{j}\right],$$

where $\eta (x_{i},t_{j})$ is the predictor term, $(\kappa_{j-1},\kappa_{j}],\;j=1,\text{…},J$ are the intervals for which the hazard rate is assumed to be constant, $\kappa_{0}=0$ and $\kappa_{J}=\max\left(t\right)$. There are different ways of choosing $t_{j},j=1,\text{…},J$, that is, the time values at which the hazard function (2) is evaluated. The two most frequently used approaches are interval end‐points $t_{j}=\kappa_{j}\;\forall \;t\in \left(\kappa_{j-1},\kappa_{j}\right]$ and interval mid‐points $t_{j}=0.5\left(\kappa_{j}+\kappa_{j-1}\right)\;\forall \;t\in \left(\kappa_{j-1},\kappa_{j}\right]$.

In order to make use of the piecewise exponential approach, it is convenient to restructure the data as outlined by Friedman (1982) and Bender et al. (2018). Therefore, let ${\tilde{T}}_{i}$ denote the true survival time (with ${\tilde{t}}_{i}$ denoting its realizations) and $C_{i}$ the (non‐informative) censoring time for subject i∈{1,…,n} such that $T_{i}:=\min\left({\tilde{T}}_{i},C_{i}\right)$ is its right‐censored time under risk with $t_{i}$ denoting its realizations. In addition, let $\delta_{i}=1\left(t_{i}={\tilde{t}}_{i}\right)$ be its non‐censoring indicator. The data is then restructured, such that for each subject i there is a row for each interval j in which it was under risk. These rows contain $t_{ij}$, an interval‐specific event indicator $\delta_{ij}$, formally being defined by

$$\delta_{ij}=\left\{\begin{array}{cc} 1\; & \text{if}\;t_{i}\in \left(\kappa_{j-1},\kappa_{j}\right]\;\text{and}\;t_{i}={\tilde{t}}_{i},\\ 0\; & \text{else,}\; \end{array}\right.$$

as well as an offset value $o_{ij}=\log\left(t_{ij}\right)$ that gives the log‐transformed time under risk and will be needed for the model estimation. This restructuring enlarges the dataset strongly and, therefore, increases the computational cost. However, since efficient estimation methods for GAMs can now be utilized, these increased computational costs are usually not a huge hurdle in practical applications of likelihood‐based estimation schemes.

Friedman (1982) proposed a linear time‐constant predictor $\eta \left(x_{i},t_{j}\right)=x_{i}^{⊤}\beta$ implying a proportional hazards model. However, this easily generalizes to more complex effects, including time‐varying effects, which implies a non‐proportional hazards model. This leads to the class of piecewise exponential additive mixed models (PAMM; Bender et al. 2018; Bender and Scheipl 2018).

### 2.2 Piecewise Exponential Additive Mixed Models

Using PAMMs, a structured additive hazard regression model of the form (1) can be constructed by deploying an additive predictor term in model (2), that is

(3) $$\eta \left(x_{i},t_{j}\right)=\sum_{k=1}^{K}f_{k}\left(x_{i},t_{j}\right),$$

leading to

$$\lambda_{i}\left(t|x_{i}\right)=\exp\left({\tilde{\lambda }}_{0}\left(t_{j}\right)+\sum_{k=1}^{K}f_{k}\left(x_{i},t_{j}\right)\right),\;\forall \;t\in \left(\kappa_{j-1},\kappa_{j}\right].$$

where $x_{i}$ denotes the covariate vector for subject i. The above notation slightly deviates from the one of Bender et al. (2018) as we do not include different effect types explicitly but implicitly as special cases of $f_{k}\left(x_{i},t_{j}\right)$. Typical examples are, among others, linear effects $f_{k}\left(x_{i},t_{j}\right)=\beta_{p}\cdot x_{ip}$, time‐constant non‐linear effects $f_{k}\left(x_{i},t_{j}\right)=f_{k}\left(x_{i}\right)$, linearly time‐varying effects $f_{k}\left(x_{i},t_{j}\right)=\beta_{p}\cdot x_{ip}\cdot t_{j}$ as well as frailty/random effects. In addition, this can be easily generalized to time‐varying covariates $x_{ipt}$. However, since time‐varying covariates are not our focus, we omit them here for notational simplicity and refer the reader to Section 3.4 of Bender et al. (2018).

In contrast to classical PEMs, for which Friedman (1982) suggested a step function as baseline hazard, Bender et al. (2018) propose to use a penalized regression spline as baseline hazard for PAMMs. This eliminates the problem of manually selecting the interval cutoff points, which is a challenging task and a common criticism of PEMs. By deploying a penalized approach, the number of cut points just needs to be large enough to provide a sufficiently good fit while overfitting is prevented due to the penalization. Hence, the standard choice of cut points for PAMMs is using all unique observed survival times. Therefore, the assumption of constant hazard rates is not very strict in practice, as it only applies for intervals, each just containing one distinct event time (i.e., ties lie in the same interval). In addition, in many applications, this makes the choice of $t_{j}$ within the interval mostly irrelevant as there are no large differences. Hence, interval end points, that is, $t_{j}=\kappa_{j}$, are often just chosen for simplicity.

### 2.3 Poisson‐Likelihood Based Inference

As mentioned before, the maximum likelihood optimization problem of a Cox‐type structured hazard regression model (SHRM; Kneib and Fahrmeir 2007), that is, of model (1), lacks a closed form. This is directly visible from its log‐likelihood, which contains an integral over the hazard rate and is given by

$$\ell_{\text{SHRM}}=\sum_{i=1}^{n}\left(\delta_{i}\left({\tilde{\lambda }}_{0}\left(t_{i}\right)+\eta \left(x_{i},t_{i}\right)\right)-\int_{0}^{t_{i}}\exp\left({\tilde{\lambda }}_{0}\left(t\right)+\eta \left(x_{i},t\right)\right)\;dt\right),$$

where $\delta_{i}$ and $t_{i}$ are defined as given in Section 2.1 and the additive predictor term $\eta (x_{i},t_{j})$ is defined according to Equation (3). In contrast, as outlined by Bender et al. (2018), the main advantage of PAMMs is that their likelihood is proportional to the one of a Poisson regression model

(4) $$E\left(\delta_{ij}|x_{i}\right)=\exp\left({\tilde{\lambda }}_{0}\left(t_{j}\right)+\eta \left(x_{i},t_{j}\right)+o_{ij}\right).$$

Hence, the PAMM and the Poisson model are equivalent with respect to their parameters and the corresponding estimation can be conducted based on the Poisson model's log‐likelihood

ℓPoisson=∑i=1n∑j=1Jilog(δij!)︸=0δij(λ∼0(tj)+η(xi,tj)+oij)−expλ∼0(tj)+η(xi,tj)+oij)−log(δij!)︸=0

where $J_{i}$ indexes the last interval in which person i is under risk, that is, $J_{i}={\mathrm{argmax}}_{j}\left\{\kappa_{j-1}:\kappa_{j-1}<t_{i}\right\}$. Therefore, the inference can be based on existing methods and one can make use of the methodological and algorithmic advances in the estimation of GAMs. This includes both, frequentist (e.g., Wood 2011) as well as Bayesian methods (e.g., Kneib et al. 2019). While the Bayesian approach may have theoretical and interpretive advantages in many cases, it is often not numerically feasible because the data transformation discussed in Section 2.1 can strongly enlarge the datasets leading to increased computational costs when relying on Markov chain Monte Carlo simulations. In contrast, when utilizing efficient frequentist estimation methods based on maximizing the likelihood (e.g., Wood 2011), the increased computational complexity is usually not of practical relevance.

Estimating the smoothing parameters is a challenging task in frequentist inference. Besides other alternatives (see, e.g., Fahrmeir et al. (2022) or Wood (2017) for an overview), Wood (2011) proposes a method utilizing a random effects perspective while avoiding the formal mixed model framework. The smoothing parameters are estimated directly from the restricted likelihood function without requiring the specification of a full mixed model structure. This is achieved by using a direct Laplace approximation that integrates out the random effects, that is, the spline coefficients. Hence, this method optimizes a well‐defined likelihood function directly with respect to the smoothing parameters. Therefore, it bypasses the need to solve mixed‐model equations.

As discussed by Wood (2017), this method is advantageous compared to smoothness selection criterion‐based and full mixed model‐based approaches in terms of convergence, precision, and numerical stability. Details about the estimation procedure as well as its asymptotic properties can be found in Wood (2011).

## 3 Capturing Heterogeneous Time‐Variation in Covariate Effects Using Functional Random Coefficients

Within the framework of PAMMs several effect types have already been introduced, including smooth, linearly time‐varying effects $t_{j}\cdot f_{k}\left(x_{ik}\right)$, linear, smoothly time‐varying effects $f_{k}\left(t_{j}\right)\cdot x_{ik}$, smooth, smoothly time‐varying effects $f_{k}\left(x_{ik},t_{j}\right)$ (see Table 3 of Bender et al. (2018) and Table 1 of Bender and Scheipl (2018) for a complete overview) as well as random effects (in terms of log‐normal frailty). However, besides Bender et al. (2019), who used a similar effect within exposure‐lag‐response association models, heterogeneously time‐varying effects of covariates have not yet been considered. They can be denoted as

(5) $$f_{kg}\left(t\right)\cdot x_{ik},$$

where g∈{1,…,G} is the deterministic explanatory grouping variable. Formally, g also has an index i but we omit that here to avoid double indices.

**TABLE 1:** Overview over the four simulation scenarios, their DGPs and the models corresponding to these DGPs.

Scenario	Effect of $x_{2}$ in the DGP	Model
(I)	Heterogeneous time‐variation	(i)
(II)	Heterogeneity & time‐variation but no interaction	(ii)
(III)	Heterogeneity only	(iii)
(IV)	Time‐variation only	(iv)

**TABLE 2:** Results for the fitted model: for the linear effects, the estimated coefficients (Coef.), the standard error (SE), and the *p*‐value, resulting from a *z*‐test, are shown. For the two smooth terms, that is, the log‐baseline f(t) and the heterogeneously time‐varying effect of FGA $f_{\text{Diagnosis}}\left(t\right)\;\cdot$ FGA, the p‐value corresponds to the test of Wood (2012). Additionally, for the smooth terms, the effective degrees of freedom (EDF) are shown. The EDF are calculated according to Wood et al. (2016).

Linear effects:	Coef.	SE	*p*‐value
Age	0.0401	0.0033	<0.0001
Sex: male	0.1481	0.0849	0.0811
Diagnosis: astrocytoma (other)	−1.4830	0.4424	0.0008
Diagnosis: mixed glioma	−0.8081	0.2617	0.0020
Diagnosis: anaplastic oligodendroglioma	−0.8842	0.2868	0.0020
Diagnosis: oligodendroglioma (other)	−1.5816	0.3347	<0.0001
Diagnosis: glioblastoma	0.9002	0.1670	<0.0001

Smooth terms:	*p*‐value	EDF
log‐baseline: f(t)	<0.0001	4.8077
FGA: $f_{\text{Diagnosis}}\left(t\right)\;\cdot$ FGA	0.0002	5.6736

**TABLE 3:** Fit measures for the proposed functional random coefficient and its competitors including a simple linear effect. The fit is measured in terms of the log‐likelihood (logLik), the integrated Brier score (IBS) and the AIC.

	logLik	IBS	AIC
Heterogeneous time‐variation	−4099.36	0.1162	8238.87
Heterogeneity and time‐variation	−4108.27	0.1174	8250.03
Heterogeneity only	−4111.04	0.1185	8250.50
Time‐variation only	−4111.00	0.1184	8251.44
Linear effect	−4111.01	0.1184	8251.20

As outlined in Section 1, we model these effects by *functional random coefficients*, which are constructed using *functional random effects* (FRE; Kneib et al. 2019) as varying coefficients. For better readability, we will leave out the effect index k for the remainder of this section.

FREs are essentially two‐dimensional anisotropic tensor product interactions, that is $f_{g}\left(t\right):=f\left(g,t\right)$, of a random effect and a smooth time effect. As it is typical for random coefficient approaches, functional random coefficients assume that the non‐linear group‐specific time‐variation is linearly interacted with the covariate $x_{ik}$, that is, they reflect the structure of effect (5).

In order to introduce anisotropic tensor product interactions formally, we first need to define the corresponding main effects $f_{1}\left(g\right)$ and $f_{2}\left(t\right)$. While denoting the nonlinear function of continuous time as $f_{2}\left(t\right)$ follows standard notation, denoting the random effect as $f_{1}\left(g\right)$ is rather uncommon. However, this notation still corresponds to classical random effects. This notation is motivated by the fact that a random effect can be constructed in terms of basis function expansion, similarly to the construction of spline effects for continuous covariates, as discussed by Kneib et al. (2019) and Hagemann et al. (2024). Hence, we can express the two main effects $f_{1}\left(g\right)$ and $f_{2}\left(t\right)$ in terms of basis function expansion as

(6) $$f_{1}\left(g\right)=\sum_{d_{1}=1}^{D_{1}}\gamma_{1d_{1}}B_{1d_{1}}\left(g\right),\;f_{2}\left(t\right)=\sum_{d_{2}=1}^{D_{2}}\gamma_{2d_{2}}B_{2d_{2}}\left(t\right),$$

where $B_{1d_{1}}\left(g\right)$ and $B_{2d_{2}}\left(t\right)$ are the basis functions, $\gamma_{1d_{1}}$ and $\gamma_{2d_{2}}$ are the basis coefficients and $D_{1}$ and $D_{2}$ are corresponding dimensions. For an introduction to univariate basis function expansions, the reader is referred to Fahrmeir et al. (2022) or Wood (2017). Their tensor product interaction is then given by

$$f\left(g,t\right)=\sum_{d_{1}=1}^{D_{1}}\sum_{d_{2}=1}^{D_{2}}\gamma_{d_{1}d_{2}}B_{d_{1}d_{2}}\left(g,t\right),$$

where the *tensor product basis functions*

$$B_{d_{1}d_{2}}\left(g,t\right)=B_{1d_{1}}\left(g\right)B_{2d_{2}}\left(t\right)$$

result from pairwise interactions of the main effect basis functions and $\gamma_{d_{1}d_{2}}$ are the *tensor product basis coefficients* which need to be estimated.

While such tensor product interactions are mainly used to construct interaction surfaces of continuous variables, we can also use them to interact smooth effects with random effects by choosing the basis functions correspondingly. Hence, we consider i.i.d. random effects, that is, log‐normal frailty, for the first main effect. As outlined by Kneib et al. (2019) and Hagemann et al. (2024), for an i.i.d. random effect, the corresponding $D_{1}=G$ basis functions are indicator functions for the group membership, that is,

$$B_{1d_{1}}\left(g\right)=1\left(g=d_{1}\right).$$

Accordingly, for the random effect, the basis function representation (6) can be rewritten as

$$f_{1}\left(g\right)=\sum_{d_{1}=1}^{D_{1}}\gamma_{1d_{1}}B_{1d_{1}}\left(g\right)=\sum_{d_{1}=1}^{G}\gamma_{1d_{1}}1\left(g=d_{1}\right)=\gamma_{1g}.$$

The time‐varying effect, that is, the second main effect, can be modeled using P‐splines (Eilers and Marx 1996). That is, using B‐spline basis functions in combination with a discrete, usually a first‐ or second‐order, penalty. The use of a cubic P‐spline, that is, deploying B‐spline basis functions of order 3, which results in a twice continuously differentiable function, has become standard. By using first order penalties, deviations from a constant are penalized across one neighboring interval in each direction. Deploying second order penalties leads to the penalization of deviations from a linear function across the next two intervals in each direction. The corresponding univariate penalty matrix of the random effect is given as a unit matrix $I_{G}$ of dimension G and that of the P‐spline as $D_{D_{2}}^{⊤}D_{D_{2}}$ where $D_{D_{2}}$ is a first or second order difference matrix of dimension $D_{2}$. Alternatively, other forms of penalized splines can be used as well, for example, thin plate splines (Wood 2003).

There are different ways of implementing penalties for tensor product interactions: it can be based on a straightforward combination of univariate penalty matrices (see Kneib et al. (2019) for such an approach). Alternatively, Wood et al. (2013) developed an approach which is not such a straightforward combination of univariate penalty matrices but is numerically advantageous, particularly when performing frequentist inference procedures and for models with random effects. This construction is based on reparameterizing the univariate smooths into fixed and random effects using an eigendecomposition of the penalty matrix. This leads to splitting the smooths into components that are not penalized, for example, constant or linear terms, and components that are subject to penalization. The model matrix for the tensor product smooth is then constructed by calculating row‐wise Kronecker products of these components. This results in each component being subject to at most one penalty which makes estimation numerically stable. For the detailed step‐wise construction procedure, see Section 3 of Wood et al. (2013).

## 4 Simulations

In this simulation study, we investigate the performance of the proposed approach with regard to two critical aspects: achieving superior fit when heterogeneous time‐variation is present and preventing overfitting in its absence.

### 4.1 Software Implementation

The data transformation discussed in Section 2.1 can be conducted in R by using the function as_ped from the package pammtools (Bender and Scheipl 2018) or, alternatively, by deploying the function survSplit from the package survival (Therneau and Grambsch 2000).

Frequentist estimation of the Poisson model is implemented in the R package mgcv: the method of Wood (2011) can be used via the function gam with method = “REML”.

The tensor product constructor proposed by Wood et al. (2013), which is discussed in Section 3, is implemented in the R package mgcv as function t2. As outlined before, this tensor product constructor is numerically advantageous over other, more straightforward approaches, such as the standard mgcv tensor product constructor te, particularly when performing frequentist inference procedures and for models with random effects. Based on t2, a straightforward implementation of the FRE is given by s(bs = “fs”). The FRE can then be deployed as a varying coefficient by linearly interacting it via the by argument, leading to a functional random coefficient. Using this implementation, we can construct a functional random coefficient for a variable x as

s(g, t, by = x, bs = “fs”, xt = list(bs = “ps”), m = c(3, 1)),

 where g is the grouping variable encoded as factor, t is the continuous time variable and a cubic P‐spline (bs = “ps”) with first order penalty is used. The first argument of m gives the degree of the P‐spline (i.e., 3 for cubic) and the second argument of m gives the order of the penalty. The full R code for the simulation and the case study is available from the GitHub repository linked in the Data Availability Statement section.

### 4.2 Data Generating Processes and Models

In order to generate survival data, both parts of the simulation study use the hazard function

(7) $$\lambda_{i}\left(t|x_{i1},x_{i2}\right)=\exp\left(3t+3x_{i1}+f\left(x_{i2},t,g\right)\right),$$

which depends on three explanatory variables: $x_{1}$, $x_{2}$, and g, the grouping variable with 4 levels. In the first part, referred to as scenario (I), heterogeneous time variation is deployed for the effect of $x_{2}$, that is, $f\left(x_{i2},t,g\right)=f_{g}\left(t\right)\cdot x_{i2}$. We then compare the fit of the model using the functional random coefficient for $x_{2}$, referred to as model (i), to the one of competing models, which are given by model (ii) including heterogeneity (as random effects) and time‐variation but not their interaction, model (iii) including only heterogeneity, and model (iv) including only time‐variation.

Hence, the three competitors contain less flexible nested effects (in the sense that model (i) can capture all effects included in the models (ii)–(iv) but not vice versa) and, therefore, are suspected to have an inferior model fit. In order to investigate the prevention of overfitting, the second part of the simulation study uses these three cases as data generating processes (DGP) and again fits the four models, leading to simulation scenarios (II)–(IV). The resulting four simulation scenarios (I)–(IV), corresponding to

(I) $\log\left(\lambda \left(t|x_{i1},x_{i2}\right)\right)=3t+3x_{i1}+f_{g}\left(t\right)\cdot x_{i2}$,
(II) $\log\left(\lambda \left(t|x_{i1},x_{i2}\right)\right)=3t+3x_{i1}+f\left(t\right)\cdot x_{i2}+\beta_{g}\cdot x_{i2}$,
(III) $\log\left(\lambda \left(t|x_{i1},x_{i2}\right)\right)=3t+3x_{i1}+\beta_{g}\cdot x_{i2}$, and
(IV) $\log\left(\lambda \left(t|x_{i1},x_{i2}\right)\right)=3t+3x_{i1}+f\left(t\right)\cdot x_{i2}$,

being the DGP, respectively, where $\beta_{g}$ denotes the random effect, are summarized in Table  1.

Figure 1 shows the effect of $x_{2}$ that is deployed in the DGP of each of the four scenarios. It can be observed that in scenario (IV) all subjects follow the curve that applies for group 1 in scenario (I). The effect of scenario (II) results from adding up $\frac{1}{2}$ of the effect of scenario (III) and $\frac{1}{2}$ of the effect of scenario (IV).

For further evaluation, the results are compared to those of an even more flexible competing model, namely a model with non‐linear group‐specific time‐covariate‐interaction, that is deploying $f\left(x_{i2},t,g\right)=f_{g}\left(t,x_{i2}\right)$ rather than $f\left(x_{i2},t,g\right)=f_{g}\left(t\right)\cdot x_{i2}$. This model can be implemented in mgcv as te(t, x, by = g, bs = c(“ps”, “ps”)). In contrast to model (i), the grouping structure for this model is introduced by just fitting the time‐covariate‐interaction for each level separately, rather than by an anisotropic random effects basis.

The simulation is conducted with three different sample sizes, n=200,400 and 800 observations, equally distributed among the four groups. This leads to 50,100 and 200 observations per group, respectively. We deploy the simulation framework suggested by Hofner et al. (2011), where the explanatory variables $x_{1}$ and $x_{2}$ are sampled from a uniform distribution, that is,

$$x_{1},x_{2}\overset{i.i.d.}{∼}U\left[0,1\right]$$

and the survival times are then sampled from model (7) using the algorithm of Bender et al. (2005). As suggested by Bender et al. (2005), censoring is introduced with exponentially distributed censoring times, leading to an average censoring rate of 10.5%. The in‐sample model fit is evaluated based on the log‐likelihood and the integrated Brier score (IBS; Graf et al. 1999), also known as the cumulative in‐sample prediction error. Hence, a smaller IBS is associated with a better model fit. The out‐of‐sample predictive accuracy is approximated based on an information criterion, namely the Akaike information criterion (AIC). The effective degrees of freedom, which are needed to compute the AIC of penalized models, are calculated according to Wood et al. (2016). All technical details of the simulation study, including the parametrization of the DGPs shown in Figure 1, are included in  Section S1 of the Supporting Information.

This simulation setup purposely introduces relatively large variation between simulation repetitions to ensure the robustness of the results. Consequently, to make differences between the models easily examinable, large effect sizes and strong time variation regarding the effect of $x_{2}$, that is, the effects shown in Figure 1, are introduced. In order to investigate whether the results are retained when using less extreme effect shapes and smaller effect sizes, which are more realistic in medical applications, an additional simulation deploying such effects is included in the Supporting Information. Besides the effects of $x_{2}$ these simulations use a similar setup as the simulation study presented in this section. Details on the DGPs are presented in Section S2 of the Supporting Information, with the effects of $x_{2}$ being shown in Figure S1.

### 4.3 Results

**FIGURE 2:**
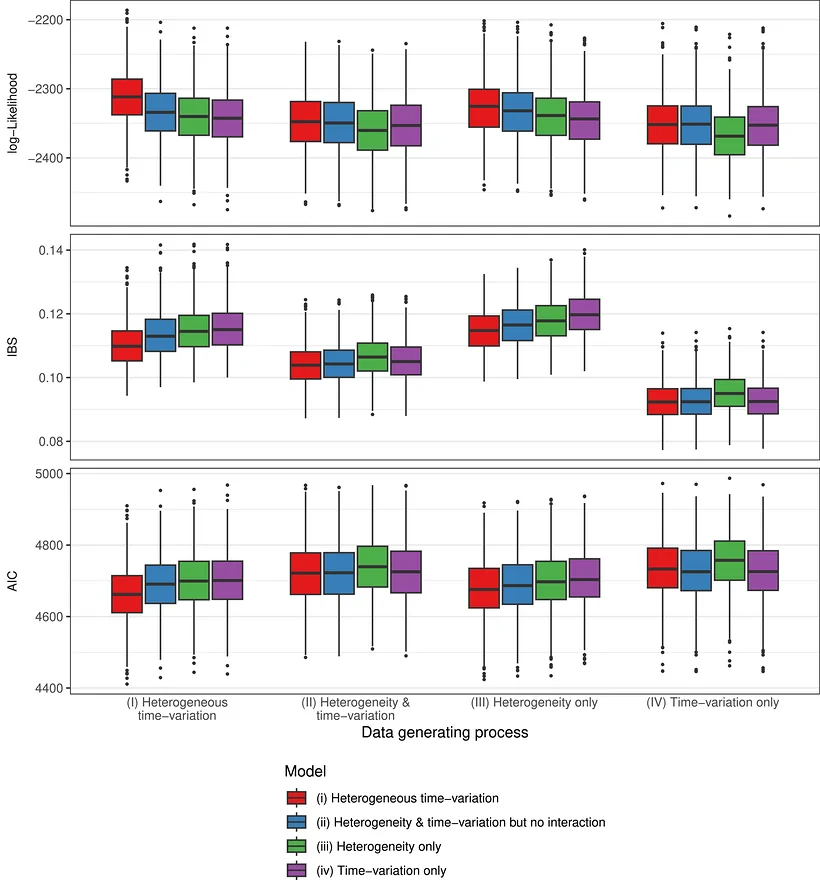
Results of the simulation study with n=400 in terms of the three fit measures. For each of the scenarios (I)–(IV) there is one block of consisting of four boxplots, one for each model (i)–(iv).

The simulation is carried out with 1000 repetitions and it can be observed that the results are almost the same across the different sample sizes. Therefore, only the results for the medium sample size, which are shown in Figure 2, are discussed here. The outcomes for the small and large sample sizes are shown in Figures S2 and S3. As discussed in Section 4.2, the simulation setup introduces relatively large variability between simulation repetitions, ensuring the robustness of the simulation results, which leads to relatively wide boxes in Figure 2.

It can be observed that, in the presence of heterogeneous time‐variation, model (i) leads to a better model fit than the less flexible approaches (ii)–(iv). In addition, the difference between model (i) and the still quite flexible model (ii) is considerably larger than the difference between model (ii) and models (iii) and (iv). This implies that modeling the effect of $x_{2}$ heterogeneously time‐varying contributes even more to the model fit than the inclusion of both main effects compared to omitting one of them. This outlines the importance of properly modeling heterogeneously time‐varying effects present in the data. The fact that this also applies with regard to the AIC implies that this might not be caused by overfitting, but by modeling the underlying DGP more accurately.

Regarding the fit of the models in scenarios (II) and (IV), we can observe the desired behavior. Here, the fit of model (i) and the model resembling the DGP, that is, model (ii) and (iv) respectively, is almost equal for all three fit measures. This strongly indicates that model (i) is penalized towards the true (less complex) model, as desired. In contrast, in scenario (III) we can observe model (i) to fit slightly better than model (iii), which resembles the underlying DGP. However, when looking at the estimated models visually (visualizations for the first 100 simulation repetitions are uploaded together with the R code), it is, with few exceptions, clearly observable that there might most likely be no time‐varying effect. Alternatively to visual inspection, formal testing could be used (e.g., based on fitting model (ii) and applying the test of Wood (2012) to the time effect), but this may not be necessary given the clear visual results. It should be noted that the problem of slight overfitting also applies for model (ii).

In Figure S4, 50 randomly selected estimated effect curves of $x_{2}$ are plotted together with the true DGP. Given the limited sample size (100 observations per group), the estimated curves appear to agree reasonably well with the actual DGPs until approximately t=0.5, with larger deviations occurring for t>0.5. This is a natural outcome for survival data, because as time progresses, most events and censorings have already occurred, resulting in the estimation being based on a decreasing amount of information.

To further investigate the model fit, we compare the difference of the fit measures of the proposed model and the model corresponding to the DGP for each simulation repetition separately. A histogram of these differences is shown in Figure S5. The ideal result would be that the penalization works so well that the curves resulting from model (i) and the model corresponding to the DGP become identical, leading to identical fit measures. While exactly identical models and fit measures may only be achieved asymptotically, for large but finite samples, we expect the differences to slightly deviate from zero with a (nearly) perfect 50:50 ratio of the sign. However, due to investigating a practically more relevant sample size (n=400), no perfect results can be expected and deviations from the perfect 50:50 split are expected. For scenario (II), it can be observed from Figure S5 that the results tend towards the desired 50:50 split, particularly for the AIC (exact result 59.3:40,7), indicating that the proposed model is effectively shrunken towards the DGP (even though not leading to perfect results due to the limited sample size). For scenario (III), that is, for time‐constant heterogeneity, slight overfitting can be observed as already discussed. For scenario (IV), a tendency towards the desired 50:50 split is visible for the log‐likelihood and the IBS, indicating that the model (i) effectively resembles the true DGP. For the AIC, a slight inferiority of the proposed model is visible, which might be caused by the model needing slightly more effective degrees of freedom to resemble the true curve than model (iv).

In Figure S6, these results are compared to those of the even more flexible competing model with non‐linear group‐specific time‐covariate‐interaction described in Section 4.2. It can be observed that, in contrast to the proposed model, the model with non‐linear group‐specific time‐covariate‐interaction leads to overfitting in all four scenarios. This is visible from, first, the log‐Likelihood and the IBS exceeding the values of the model resembling the corresponding DGP and, second, the AIC indicating an inferior out‐of‐sample fit. Hence, in contrast to the model proposed in this paper, the additional flexibility of this competitor seems to cause overfitting. Causing overfitting, even though the model deploys a penalized estimation, may indicate that, due to the higher model complexity, the sample size is not large enough to ensure effective penalization. In addition, the computational costs of this model strongly exceed those of the model deploying the functional random coefficient.

We investigate the robustness of the presented results with regard to unequal group sizes by performing additional simulations in which the n=400 observations were distributed unevenly across the four groups, resulting in group sizes of 200, 100, 50, and 50. The results are shown in Figure S7. It can be observed that the results only slightly deviate from the ones observed for equal group sizes and, therefore, imply the same conclusions. For these unequal group sizes, Figure S8 shows 50 randomly selected estimated effect curves of $x_{2}$, corresponding to the same simulation seeds as those shown in Figure S4. Compared to Figure S4, the results look overall very similar and it seems there are no systematic deviations. This supports our conclusions from Figure S7. Hence, we conclude that the proposed method is robust against unequal group sizes.

As discussed in Section 4.2, the DGPs investigated in this section imply both large effect sizes and strong time variation. While this was purposely chosen to make differences between the models easily examinable, results for less extreme effect shapes and smaller effect sizes, which are more realistic in medical applications, are presented in Figures S9 and S10. The reduced effect size, together with the large variability between the simulation repetitions, leads to differences in the fit measures being barely visible in Figure S9. However, Figure S10, which shows the difference between the model corresponding to the DGP and the proposed model (i) for each simulation repetition separately, is consistent with what was observed from Figure S5, although on a smaller scale. Hence, we conclude that this section's results hold with smaller effect sizes and less extreme effect shapes.

In conclusion, the simulation study shows that the proposed functional random coefficient approach can flexibly capture heterogeneous time‐variation within the covariate effects. In addition, if the time‐variation is homogeneous, our proposed model does not lead to overfitting due being penalized towards the true model. Only if time‐variation is fully absent, slight overfitting can be observed. However, in these cases, it is usually easy to visually recognize that there might not be any time variation in the data. Therefore, during model selection, one should always visually inspect whether there is time variation in the data at all. These results are retained for unequal group sizes, smaller effect sizes and less extreme effect shapes. Compared to an even more flexible competing model with non‐linear group‐specific time‐covariate‐interaction, the proposed model reduces overfitting and computational costs.

## 5 Brain Tumor Case Study

We apply the proposed approach to a brain tumor survival example based on data from Ceccarelli et al. (2016). This dataset includes patients with a glioma divided into five different diagnoses: anaplastic astrocytoma, astrocytoma (other), anaplastic oligodendroglioma, oligodendroglioma (other), glioblastoma and mixed glioma. After removing 37 patients due to missing values, the total number of participants is n=1094, of which 593 are non‐censored and 501 are censored. Survival times were recorded exact to the day and for 3 persons, who died already on their admission day, the survival time is set to half a day. We introduce an end‐of‐study, that is, an administrative censoring, after 8 years because only 28 patients remain at this time. In addition to the five diagnoses, the age and sex of the patients and *fraction genome altered* (FGA) are recorded. In the Supporting Information, Figure S11 shows the corresponding Kaplan–Meier curves, Figure S12 presents the empirical distribution of the event times in terms of a histogram, and Table S1 summarizes descriptive statistics for the explanatory variables.

The FGA is commonly used in cancer research and represents the proportion of a tumor's genome that is affected by gains or losses of DNA segments, for example, amplifications or deletions. Previous studies (see, e.g., Mehta et al. 2005) show that a higher FGA can be an indicator for aggressive tumor behavior and Dhital and Rodriguez‐Bravo (2023) even state that a high FGA is an independent predictor for a reduced overall survival.

We suspect that the effect of the FGA might be time‐varying and diagnosis‐specific and that the time‐variation might also be heterogeneous between diagnoses. Therefore, we model this effect by a functional random coefficient. This leads to the final model being a PAMM with a P‐spline based baseline hazard, the diagnosis‐specifically time‐varying effect of FGA, a linear effect for sex and age and a fixed effect for the diagnosis. For both, the log‐baseline hazard and the functional random effect, we choose cubic P‐splines with first order penalty and 9 inner knots, such that each of the intervals corresponds to 1 year.

The estimated regression coefficients are shown in Table 2. A higher age significantly increases the hazard rate, which is an expected result as age usually increases the risk of death. In contrast, the effect of sex is not significant at a 5%‐level. Compared to the anaplastic astrocytoma, which is the reference category, all other diagnoses have a significant effect on the hazard function. While the risk of death is increased for the glioblastoma, it is reduced for the other three diagnoses.

**FIGURE 3:**
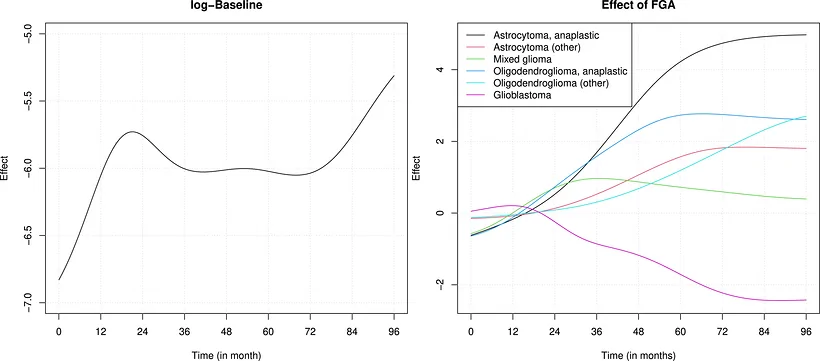
Estimated Smooth effects for the log‐baseline function and the functional random coefficient of FGA.

The non‐linear baseline hazard as well as the effect of the FGA is shown in Figure 3. Both effects are significant with regard to the test of Wood (2012), meaning that we can reject the null hypotheses of f(t)=0 and $f_{\text{Diagnosis}}\left(t\right)=0$. The baseline hazard increases nearly linearly for the first year and a half, then decreases slightly for another year and a half, and then remains nearly constant for the next 3 years before increasing again. The local maximum of the baseline hazard between 18 and 24 months seems surprising at first sight. However, when investigating the Kaplan–Meier curves and the event time histogram (see Figures S11 and S12), it appears that this is not an artifact of the model, but reflects the underlying raw data. Such an effect could have various causes, in particular unobservable covariates. The model's ability to capture unexpected effects in the underlying raw data is a strong advantage of the penalized spline‐based approach.

With regard to the effect of FGA, our main focus, we can indeed observe that the effect of FGA strongly varies over time as well as between the diagnoses and that the time variation is also quite different between the diagnoses. While there are no major differences in the first year and a half, the curves differ considerably thereafter. While the effect decreases over time for glioblastoma, it increases for the other four diagnoses. Hence, these effects might cancel out if the time‐variation is not modeled diagnosis‐specific. This outlines the practical relevance of our approach.

We compare the model fit to the competitors introduced in Section 4 as well as a model deploying a simple linear effect of FGA, which is shown in Table  3. It can be observed that modeling the time‐varying effect of FGA diagnoses specifically leads to the best model fit. This also agrees with the visual impression from Figure 3. Including only random effects or only time variation does not lead to a notable increase in model fit compared to the linear effect. In addition, even the model including heterogeneity as well as time‐variation only slightly increases the model fit compared to the increase that is achieved by using the functional random coefficient. This coincides with the impression from Figure 3, indicating that the time‐variation might cancel out if it is not modeled diagnoses specifically. The fact that this also applies to the AIC indicates that this does not result from overfitting.

In conclusion, in this application, the use of a functional random coefficient allowed us to capture diagnosis‐specific time variation in the effect of FGA on survival. In addition, the individual curves shown in Figure 3 can improve the understanding of FGA as a predictor of survival.

## 6 Conclusion

In this paper, we introduced heterogeneously time‐varying covariate effects to hazard regression models. This provides an appropriate model for cases in which the effect of a covariate is not only time‐varying and subgroup‐specific but its time‐variation is subgroup‐specific, too.

The proposed method makes use of the existing framework of PAMMs which enables us to deploy an efficient Poisson model‐based inference. Our approach allows for non‐linear time‐effects due to being based on penalized splines and uses an efficient random effects basis to model the heterogeneity. In addition, the penalization mostly prevents our method from overfitting in absence of heterogeneous time‐variation. The corresponding simulation study only shows slight overfitting if time‐effects are fully absent. However, it is easy to visually assess such cases as outlined in Section 4.3. On the other hand, in presence of heterogeneous time‐variation, the simulation study outlines the superior fit of our approach.

We apply this model to a brain tumor case study. Here, the effect of the FGA varies over time and this time‐variation is highly diagnosis‐specific. Therefore, modeling FGA with a diagnosis‐specific time‐varying effect not only greatly improves the model fit, but also prevents the effects from canceling each other out. This provides additional interpretability and may lead to a better understanding of FGA as a risk predictor. Thus, this case study outlines the practical relevance of the proposed method.

Future possible research includes introducing this type of effect to Bayesian survival models. This is mainly a matter of computational efficiency since in a Bayesian setting, for both—the piecewise exponential approach and the direct approach involving an integral over the hazard rate—estimating a FRE might lead to very high computational costs. Further research is also needed to investigate whether the penalized structure of the presented approach could also be a (partial) solution to the problem that an incorrect functional form can give the appearance of non‐proportional hazards reported by Abrahamowicz and MacKenzie (2007). In addition, other applications, such as use in multicenter studies, should be explored.

## Funding

This work has been supported by the Research Training Group ”Biostatistical Methods for High‐Dimensional Data in Toxicology“ (RTG 2624, P7) funded by the Deutsche Forschungsgemeinschaft (DFG, German Research Foundation, Project Number 427806116).

## Conflicts of Interest

The authors declare no conflicts of interest.

## Open Research Badges

This article has earned an Open Data badge for making publicly available the digitally‐shareable data necessary to reproduce the reported results. The data is available in the Supporting Information section.

This article has earned an open data badge “**Reproducible Research**” for making publicly available the code necessary to reproduce the reported results. The results reported in this article could fully be reproduced.

## Supporting information


**Supporting File 1:** bimj70179‐sup‐0001‐SuppMat.pdf.

**Supporting File 2:** bimj70179‐sup‐0002‐Data.zip.

## Data Availability

The R code and data necessary to reproduce the reported results, figures, and tables are available in the Supporting Information. All R functions are also available at https://github.com/Niklas191/heterogeneous_time‐variation. The current version of the case study data set is publicly available at the cBioPortal database with IDs gbm_tcga_gdc and difg_tcga_gdc.
