# Supplementary material for: Capturing Heterogeneous Time‐Variation in Covariate Effects in Non‐Proportional Hazard Regression Models
Source: Biom J. 2026 Sep 20;68(5):e70179. doi: 10.1002/bimj.70179 (PMC13590908; doi:10.1002/bimj.70179)
Supplement: Supplementary file 1 [file BIMJ-68-e70179-s002.pdf]

Capturing heterogeneous time-variation in covariate effects in non-proportional hazard regression models

Supporting information

Niklas Hagemann[1,2], Thomas Kneib[3] and Kathrin Möllenhoff[1,2]

[1] Institute of Medical Statistics and Computational Biology, Faculty of Medicine, University of Cologne, Germany

[2] Division of Mathematics, Department of Mathematics and Computer Science, University of Cologne, Germany

[3] Chair of Statistics and Campus Institute Data Science, Georg August University Göttingen, Germany

S 1 Technical details of the simulation study

The data generating processes of the simulation study are defined by the hazard function

$$\lambda_i(t|x_{1i}, x_{2i}) = \exp\left\{3t + 3x_{i1} + f(x_{i2}, t, g)\right\},$$

where for the effect of x_{i2} in the four scenarios (I) - (IV), the corresponding functions

$$f^{(\text{I})}(x_{i2}, t, g) = \begin{cases} (0.5 + 18t - 48t^2 + 32t^3)x_{i2} & \text{if } g = 1, \\ (2.5 - 30.667t + 137.33t^2 - 213.3t^3 + 107t^4)x_{i2} & \text{if } g = 2, \\ (0.5 - 8.7t + 54.47t^2 - 81.6t^3 + 37t^4)x_{i2} & \text{if } g = 3, \\ (1.5 + 6.267t - 38.73t^2 + 58.1t^3 - 27t^4)x_{i2} & \text{if } g = 4, \end{cases}$$

and

$$f^{(\text{II})}(x_{i2}, t, g) = 0.5f^{(\text{III})}(x_{i2}, t, g) + 0.5f^{(\text{IV})}(x_{i2}, t, g) = \begin{cases} 0.5(1 + 18t + 46t^2 + 32t^3)x_{i2} & \text{if } g = 1, \\ 0.5(1.5 + 18t + 46t^2 + 32t^3)x_{i2} & \text{if } g = 2, \\ 0.5(2 + 18t + 46t^2 + 32t^3)x_{i2} & \text{if } g = 3, \\ 0.5(2.5 + 18t + 46t^2 + 32t^3)x_{i2} & \text{if } g = 4, \end{cases}$$

and

$$f^{(\text{III})}(x_{i2}, t, g) = \begin{cases} 0.5x_{i2} & \text{if } g = 1, \\ x_{i2} & \text{if } g = 2, \\ 1.5x_{i2} & \text{if } g = 3, \\ 2x_{i2} & \text{if } g = 4, \end{cases}$$

and

$$f^{(\text{IV})}(x_{i2}, t, g) = (0.5 + 18t + 46t^2 + 32t^3)x_{i2}$$

are deployed.

The distribution of the censoring times is given by

$$C_i \overset{i.i.d.}{\sim} Exp(0.4).$$

S 2 Technical details of the second simulation study with less extreme effect shapes and smaller effect sizes, which are more realistic in medical applications

The data generating processes of the simulation study are defined by the hazard function

$$\lambda_i(t|x_{1i}, x_{2i}) = \exp\left\{2 + 0.3t + 0.3x_{i1} + f(x_{i2}, t, g)\right\},$$

where the effects of x_{i2} in the four scenarios (I) - (IV) are shown in Figure 1 and correspond to the functions

$$f^{(\mathrm{I})}(x_{i2}, t, g) = \begin{cases} 0.9\,\frac{t^9}{0.35^9+t^9}\,x_{i2} & \text{if } g = 1, \\ 0.8\,\frac{t^7}{0.6^7+t^7}\,x_{i2} & \text{if } g = 2, \\ 1.1\,\frac{t^{1.8}}{0.1^{1.8}+t^{1.8}}\,x_{i2} & \text{if } g = 3, \\ 0.03\,(\exp(\frac{t}{0.3}) - 1)\,x_{i2} & \text{if } g = 4, \end{cases}$$

and

$$f^{(\mathrm{II})}(x_{i2}, t, g) = 0.5f^{(\mathrm{III})}(x_{i2}, t, g) + 0.5f^{(\mathrm{IV})}(x_{i2}, t, g) = \begin{cases} 0.5\,(0.25 + 0.9\,\frac{t^9}{0.35^9+t^9})x_{i2} & \text{if } g = 1, \\ 0.5(0.5 + 0.9\,\frac{t^9}{0.35^9+t^9})x_{i2} & \text{if } g = 2, \\ 0.5(0.75 + 0.9\,\frac{t^9}{0.35^9+t^9})x_{i2} & \text{if } g = 3, \\ 0.5(1 + 0.9\,\frac{t^9}{0.35^9+t^9})x_{i2} & \text{if } g = 4, \end{cases}$$

and

$$f^{(\mathrm{III})}(x_{i2}, t, g) = \begin{cases} 0.25\,x_{i2} & \text{if } g = 1, \\ 0.5\,x_{i2} & \text{if } g = 2, \\ 0.75\,x_{i2} & \text{if } g = 3, \\ x_{i2} & \text{if } g = 4, \end{cases}$$

and

$$f^{(\mathrm{IV})}(x_{i2}, t, g) = 0.9\,\frac{t^9}{0.35^9 + t^9}\,x_{i2},$$

respectively.

The distribution of the censoring times is given by

$$C_i \overset{i.i.d.}{\sim} Exp(0.4).$$

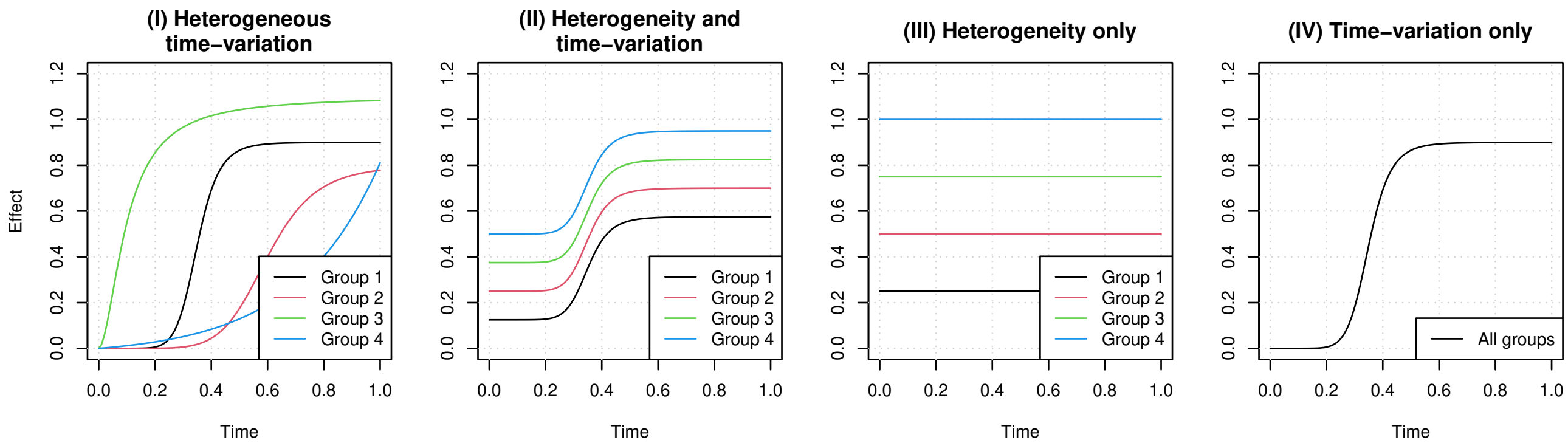

Figure S 1: Effect of x_2 in the DGPs of the four scenarios of the second simulation study.

S 3 Additional plots and tables

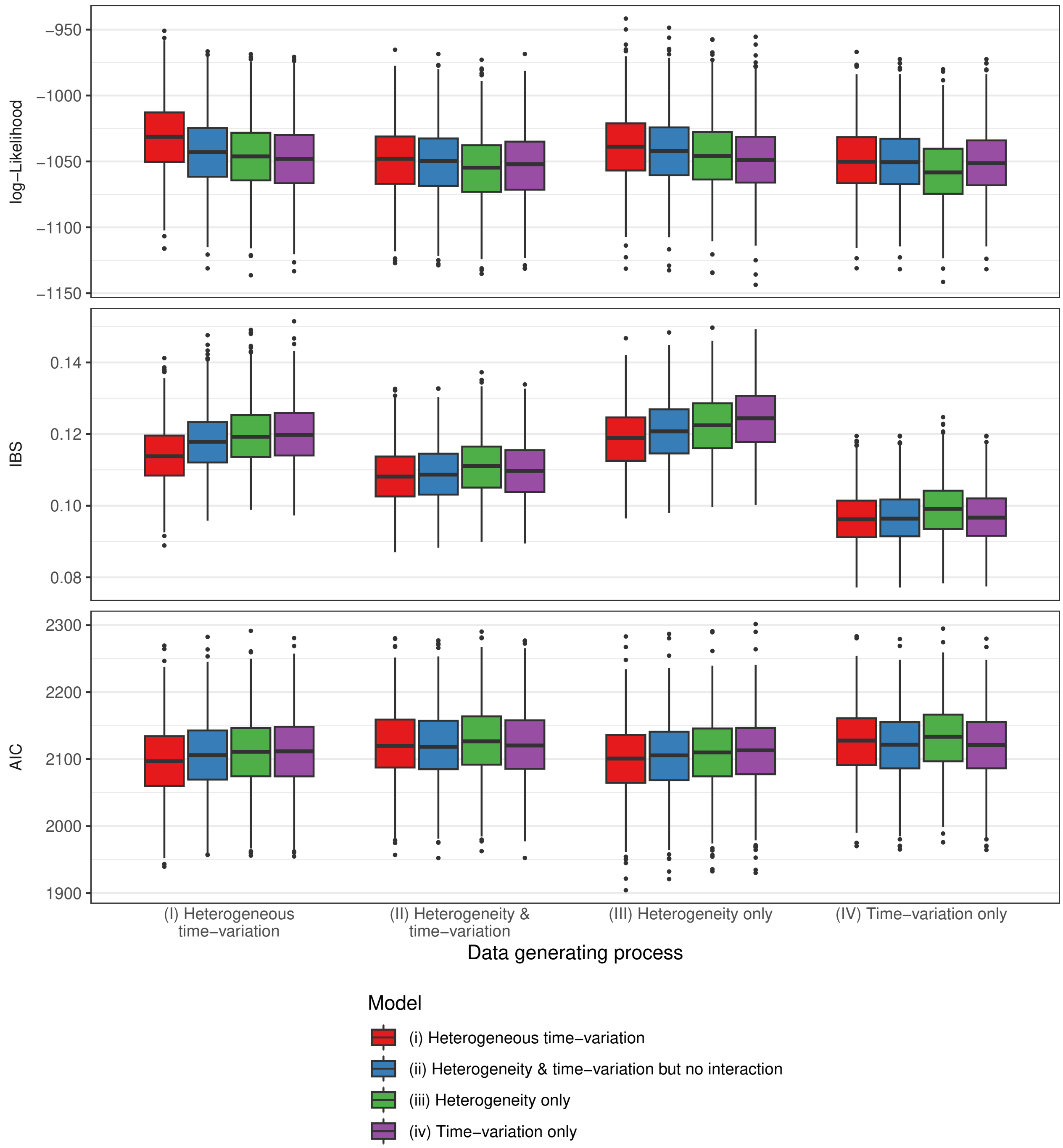

Figure S 2: Results of the simulation study with $n = 200$ in terms of the three fit measures. For each of the scenarios (I) - (IV) there is one block consisting of four boxplots, one for each model (i) - (iv).

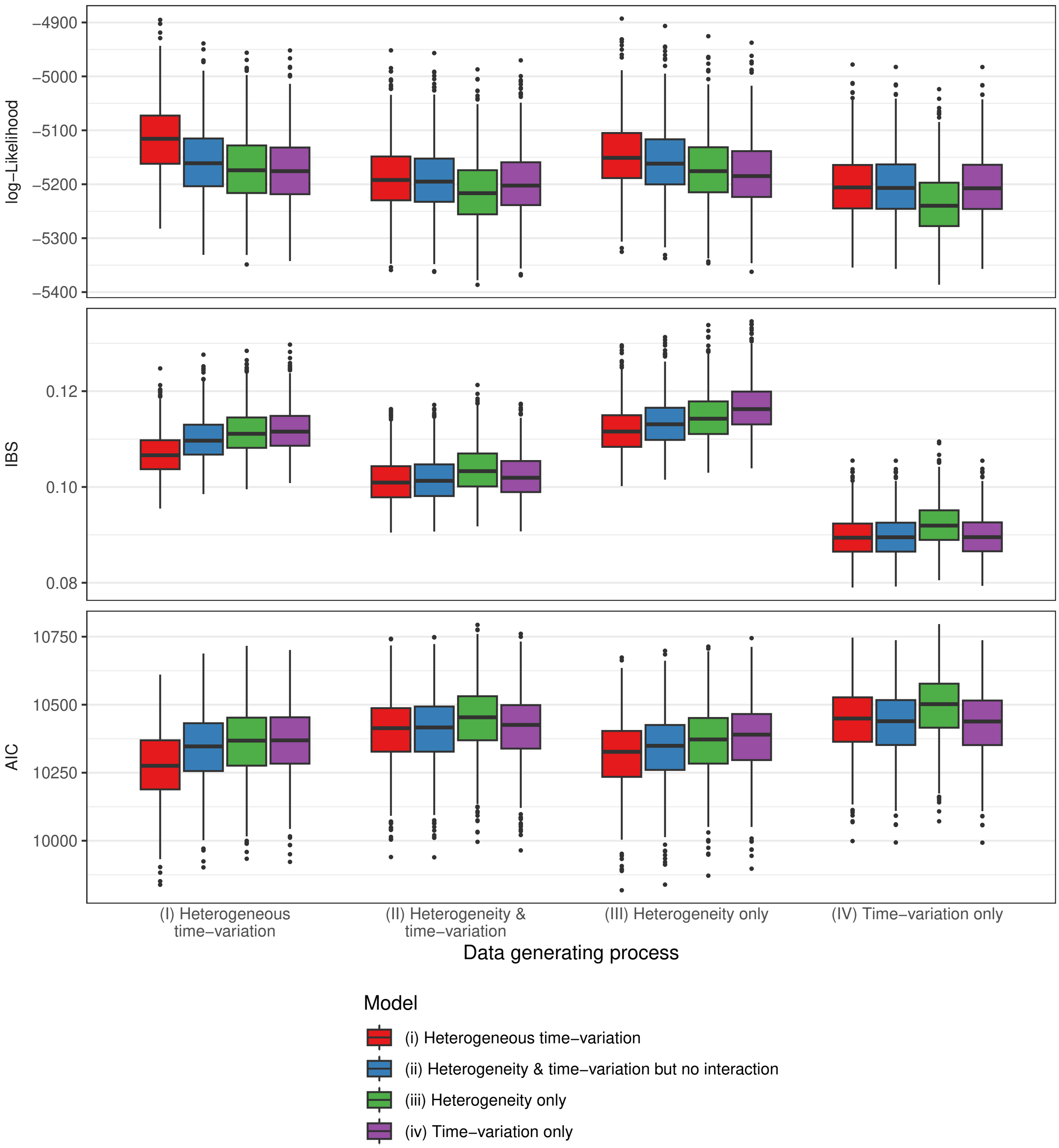

Figure S 3: Results of the simulation study with $n = 800$ in terms of the three fit measures. For each of the scenarios (I) - (IV) there is one block consisting of four boxplots, one for each model (i) - (iv).

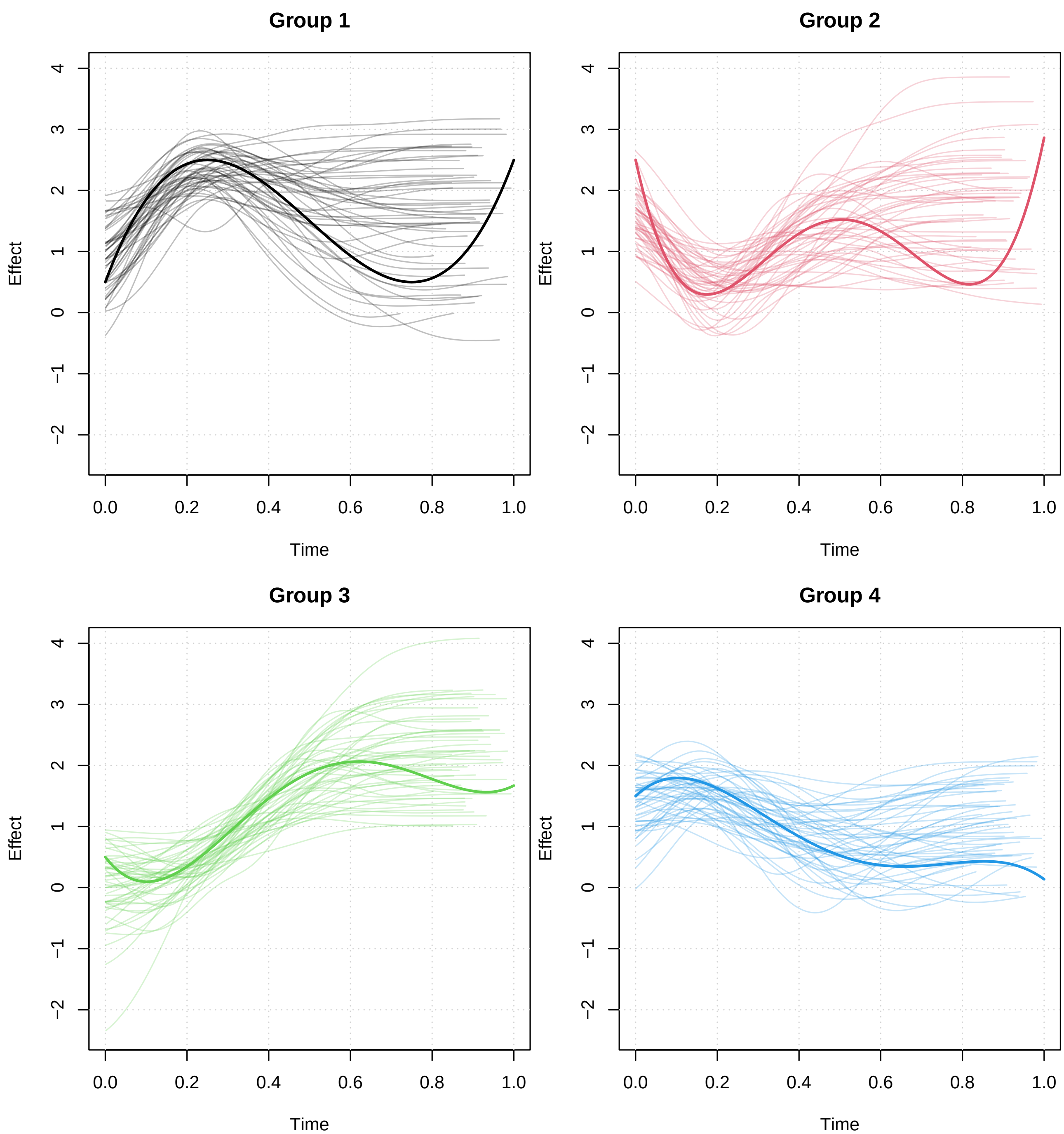

Figure S 4: Plot of the fitted curves $\hat{f}_g(t)$ for 50 randomly selected simulation repetitions. The thick solid curve represents the DGPs, and the thinner semi-transparent curves show the estimated curves.

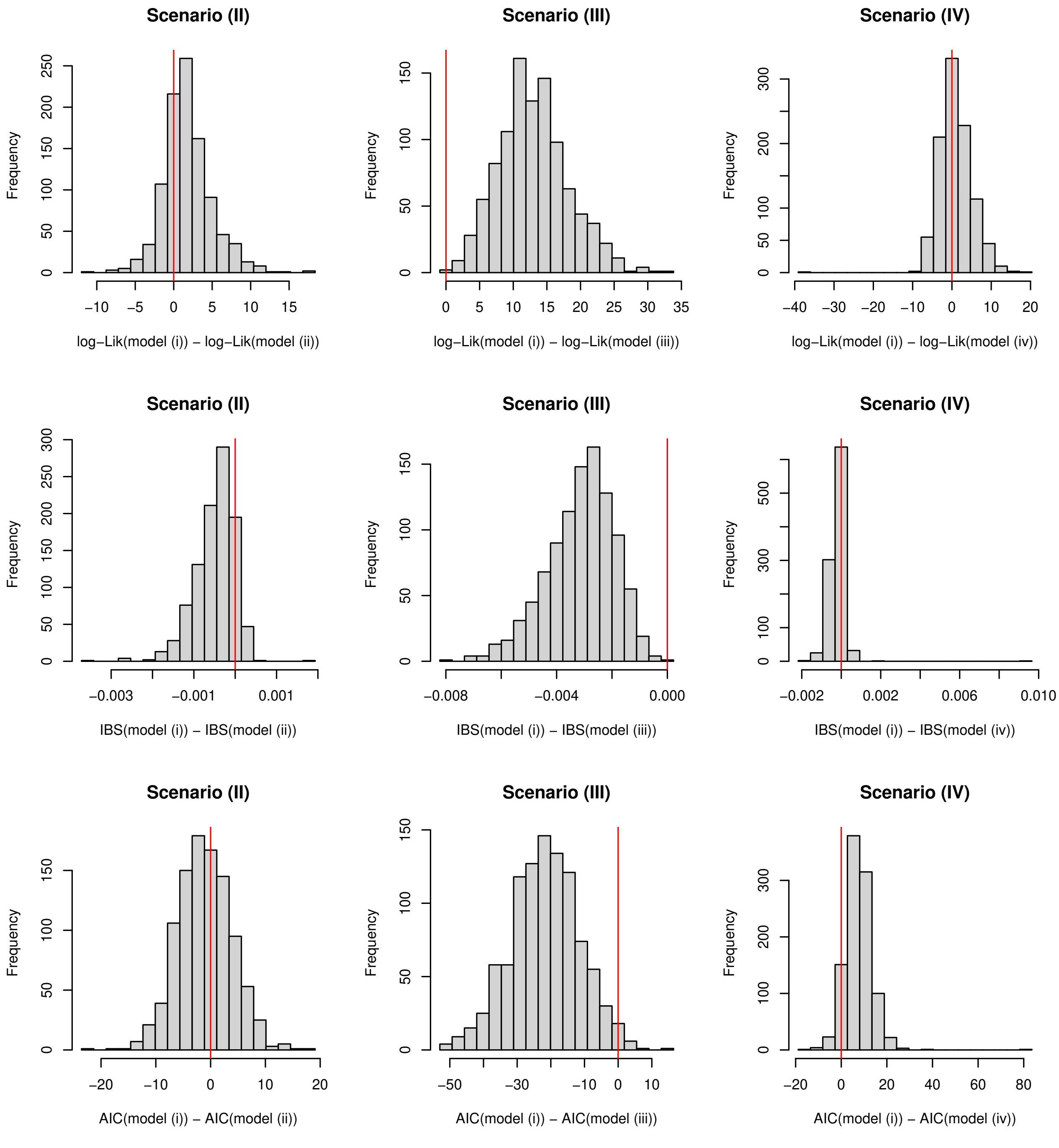

Figure S 5: Histograms of the differences between the fit measures of the proposed model and the model corresponding to the DGP for each simulation repetition. The red line corresponds to zero.

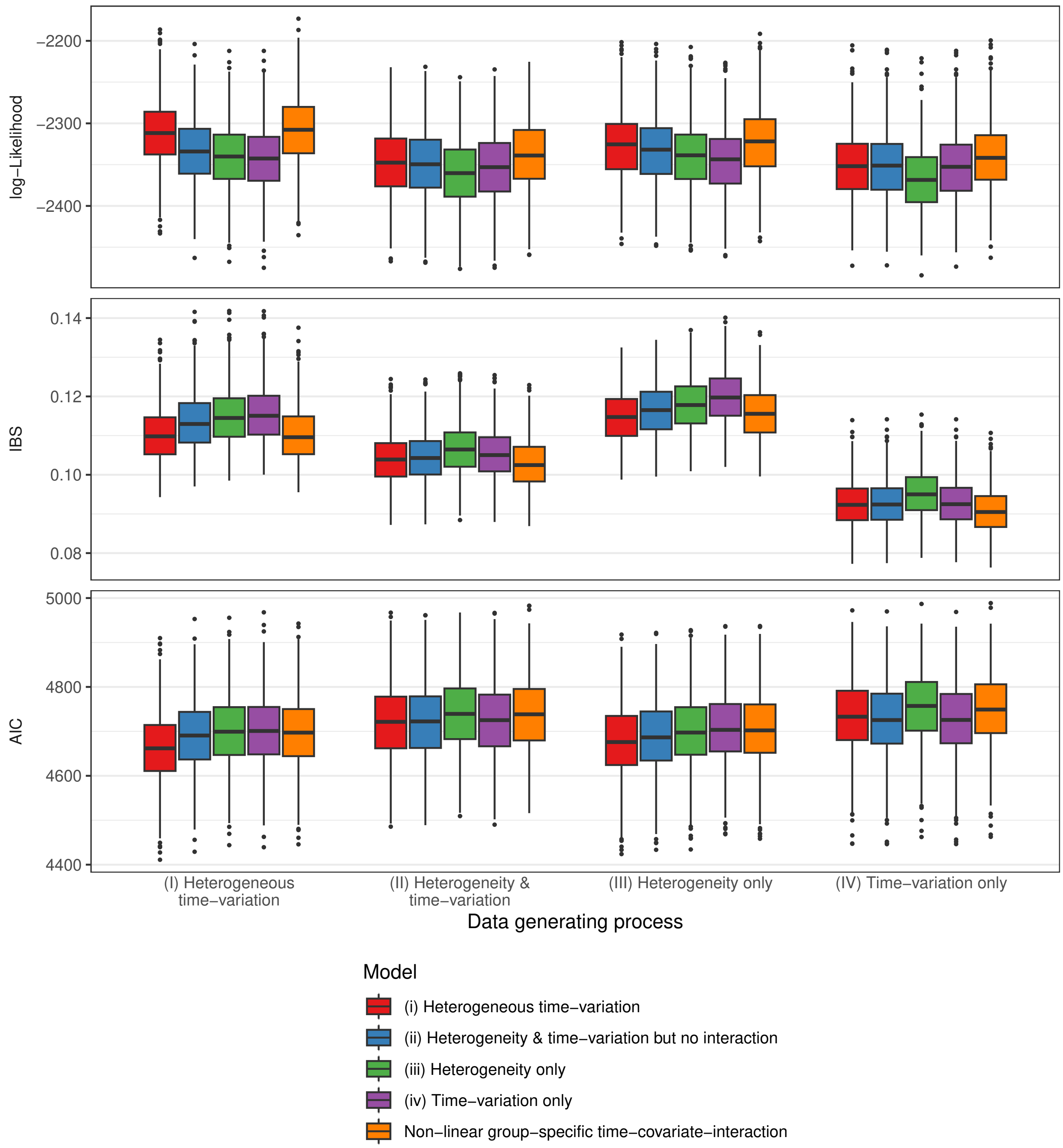

Figure S 6: Comparison of the results of the simulation study with $n = 400$ with an additional model introducing non-linear group-specific time-covariable interaction via `te(t, x₂, by = g)`. For each of the scenarios (I) - (IV) there is one block consisting of five boxplots, one for each model (i) - (iv) and one for the additional model.

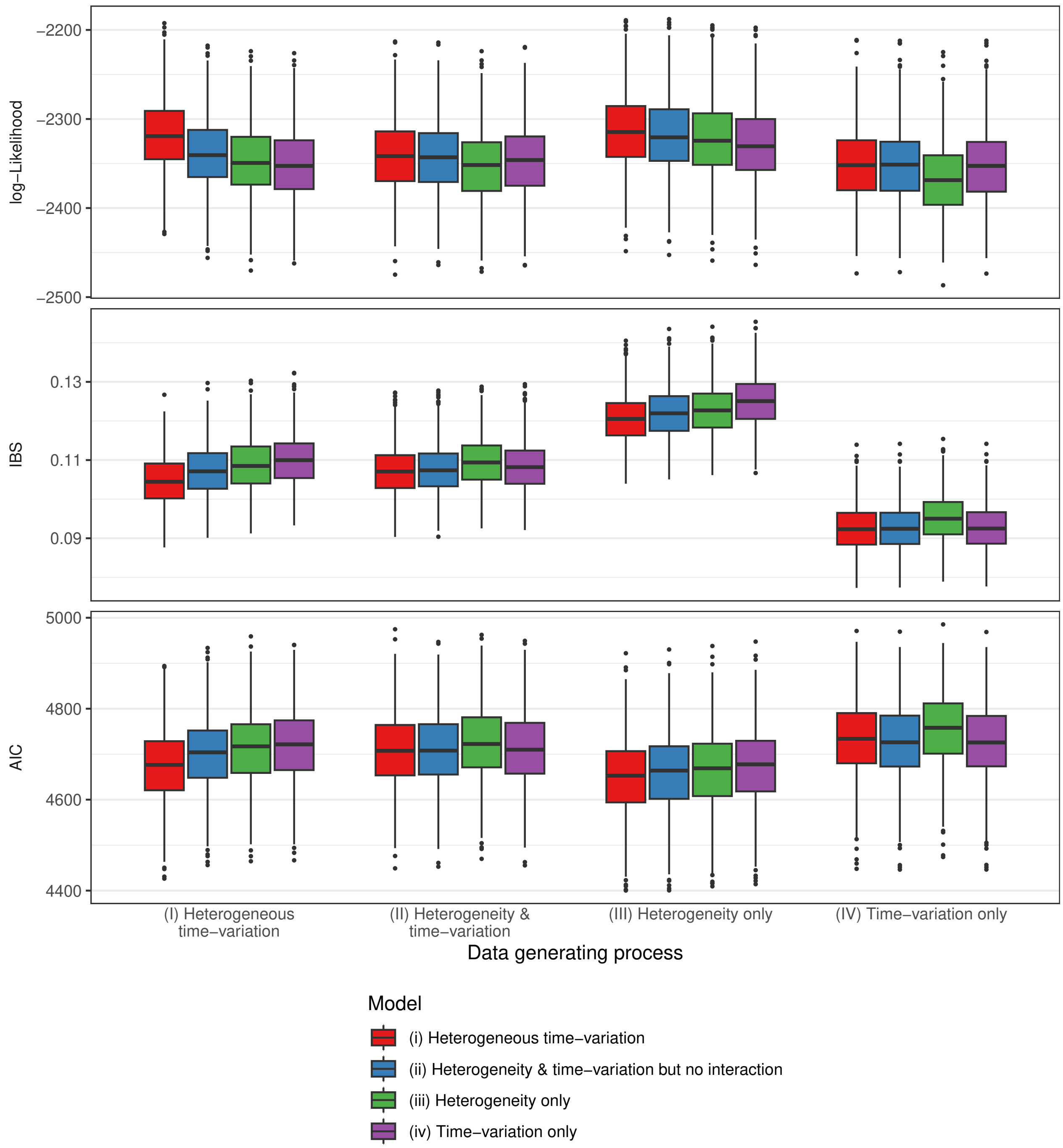

Figure S 7: Results of the simulation study with unequal group sizes $(n_1, n_2, n_3, n_4) = (200, 100, 50, 50)$ in terms of the three fit measures. For each of the scenarios (I) - (IV) there is one block consisting of four boxplots, one for each model (i) - (iv).

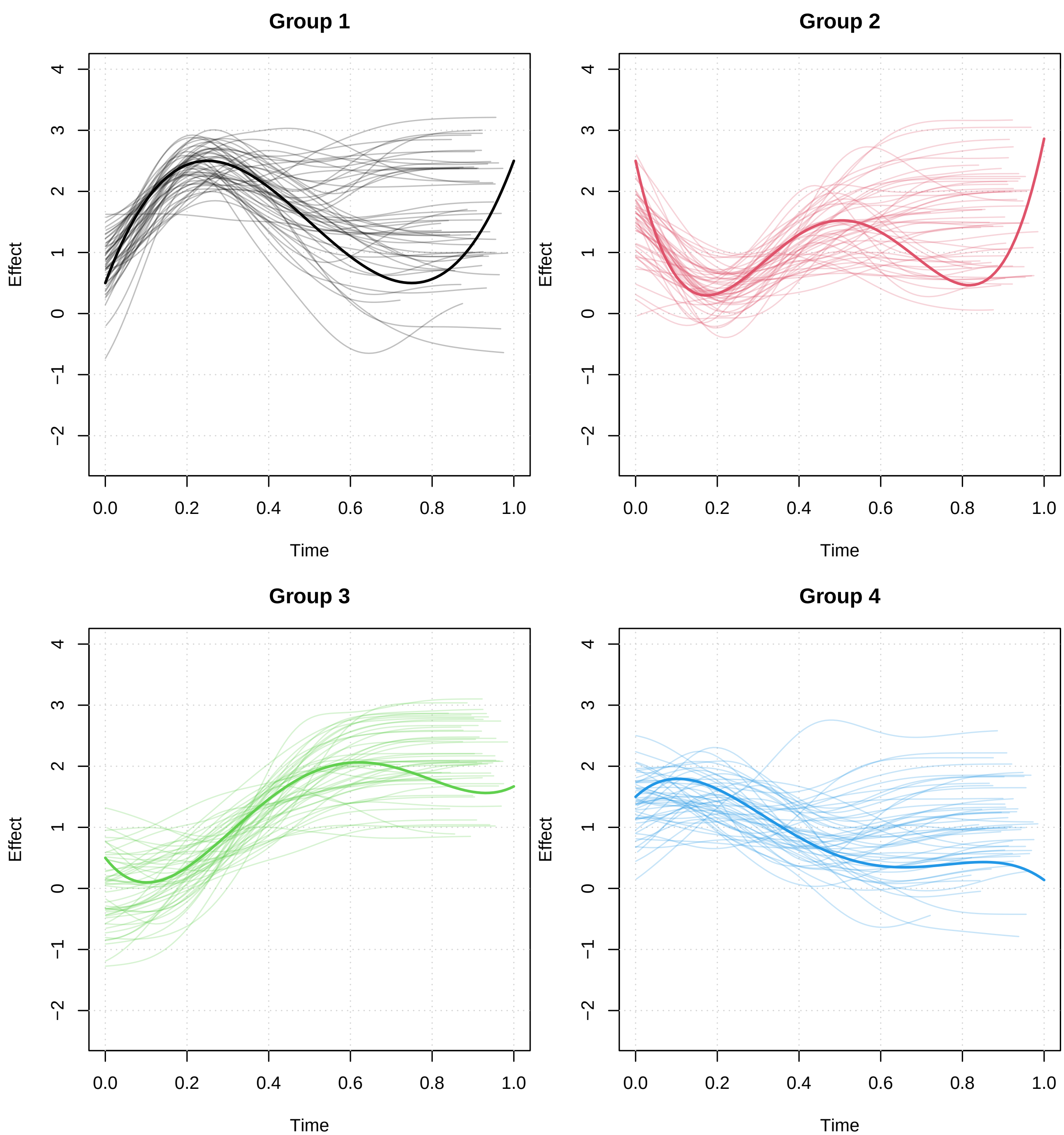

Figure S 8: Plot of the fitted curves $\hat{f}_g(t)$ for 50 randomly selected simulation repetitions of the simulation study with unequal group sizes $(n_1, n_2, n_3, n_4) = (200, 100, 50, 50)$. The thick solid curve represents the DGPs, and the thinner semi-transparent curves show the estimated curves.

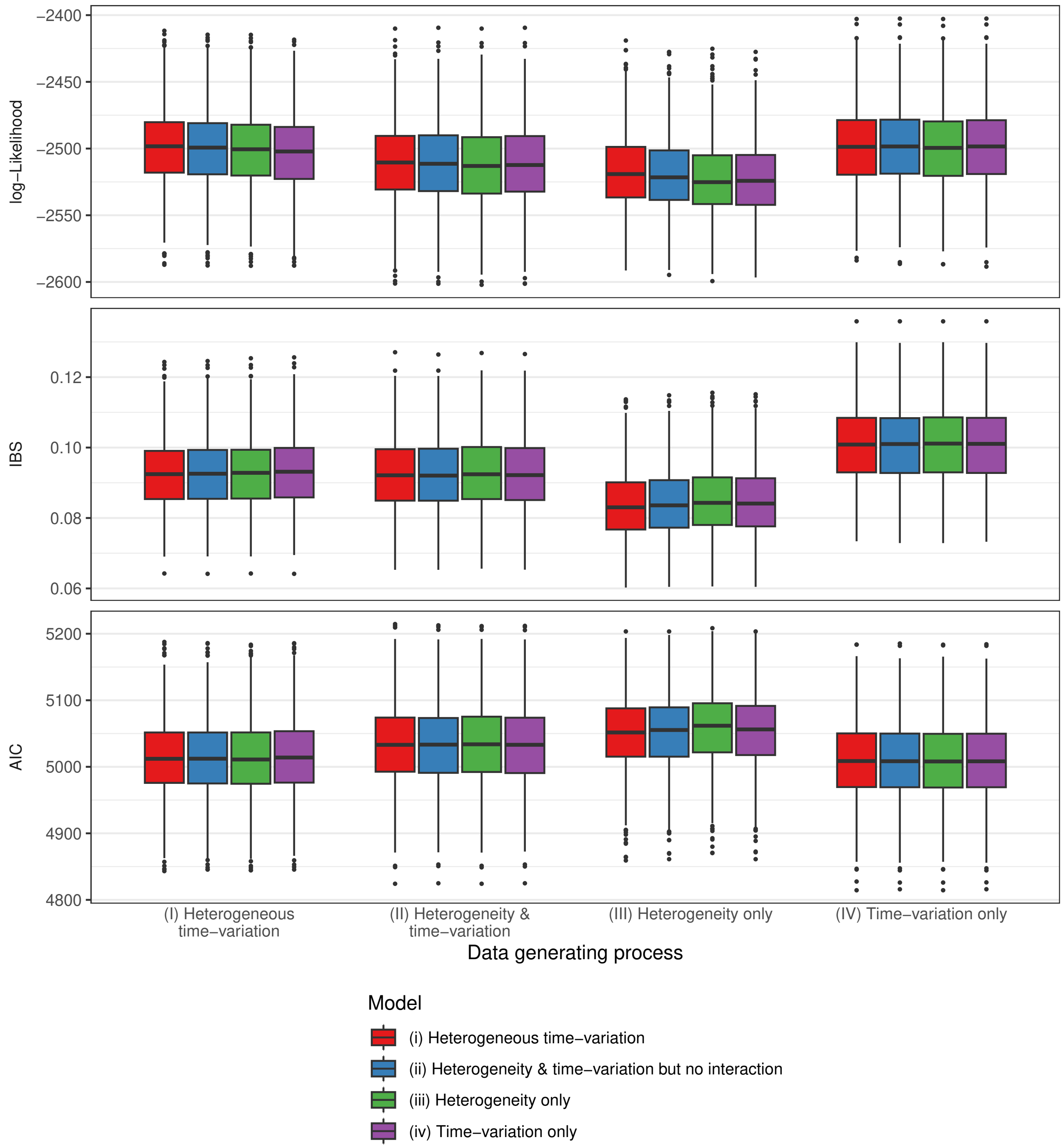

Figure S 9: Results of the additional simulation study presented in Section S 2 in terms of the three fit measures. For each of the scenarios (I) - (IV) there is one block consisting of four boxplots, one for each model (i) - (iv).

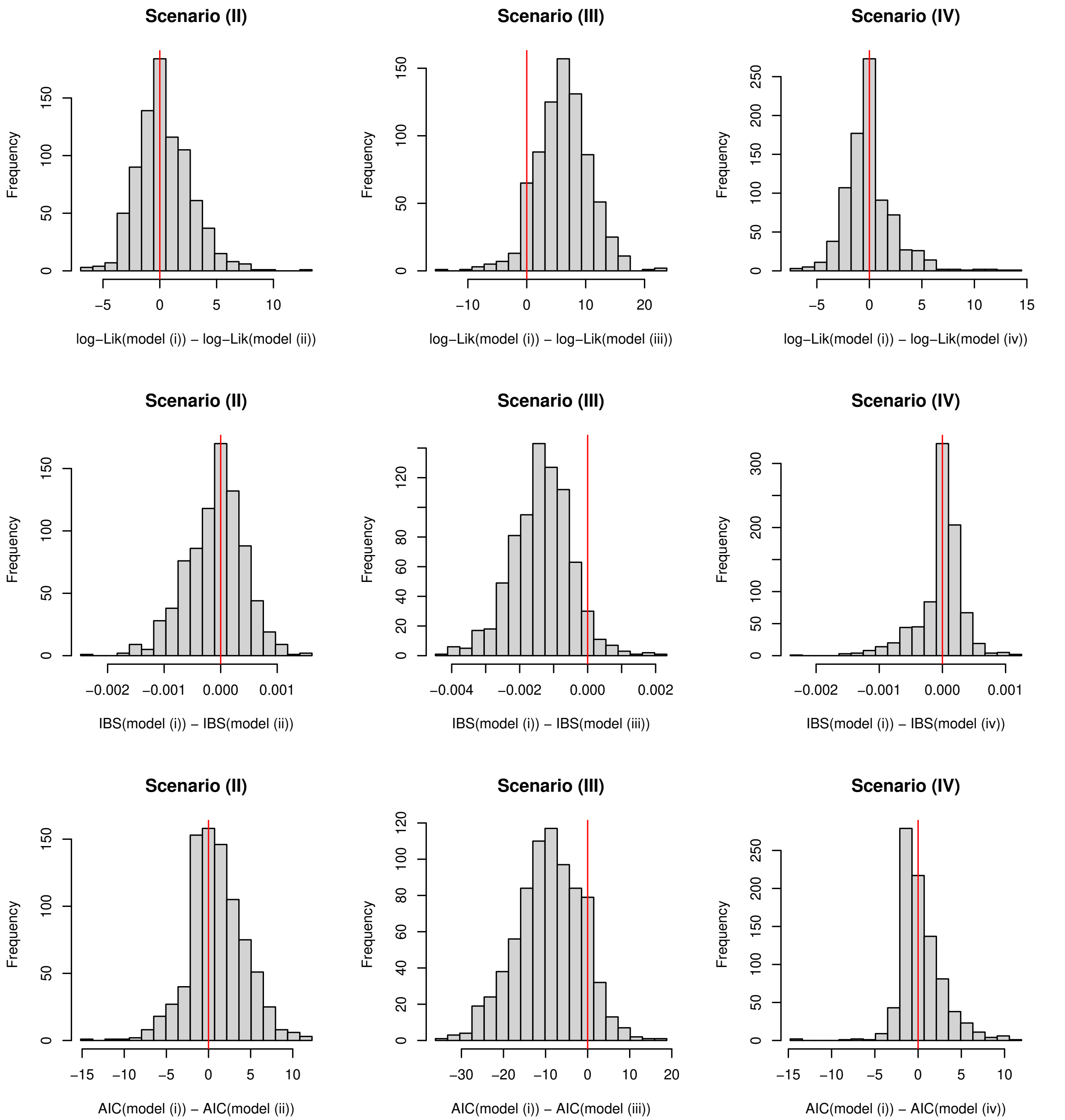

Figure S 10: Histograms of the differences between the fit measures of the proposed model and the model corresponding to the DGP for each simulation repetition of the additional simulation study presented in Section S 2. The red line corresponds to zero.

S 1: Descriptive statistics of the explanatory variables for the case study dataset. For the categorical variables, the counts for the corresponding categories are provided. For the continuous variables mean and standard deviation (SD) are given.

		Count	Mean	SD
Diagnosis	Anaplastic astrocytoma	130		
	Astrocytoma (other)	64		
	Mixed glioma	130		
	Anaplastic oligodendroglioma	78		
	Oligodendroglioma (other)	108		
	Glioblastoma	584		
Sex	Female	455		
	Male	639		
Age			50.979	15.747
FGA			0.165	0.121

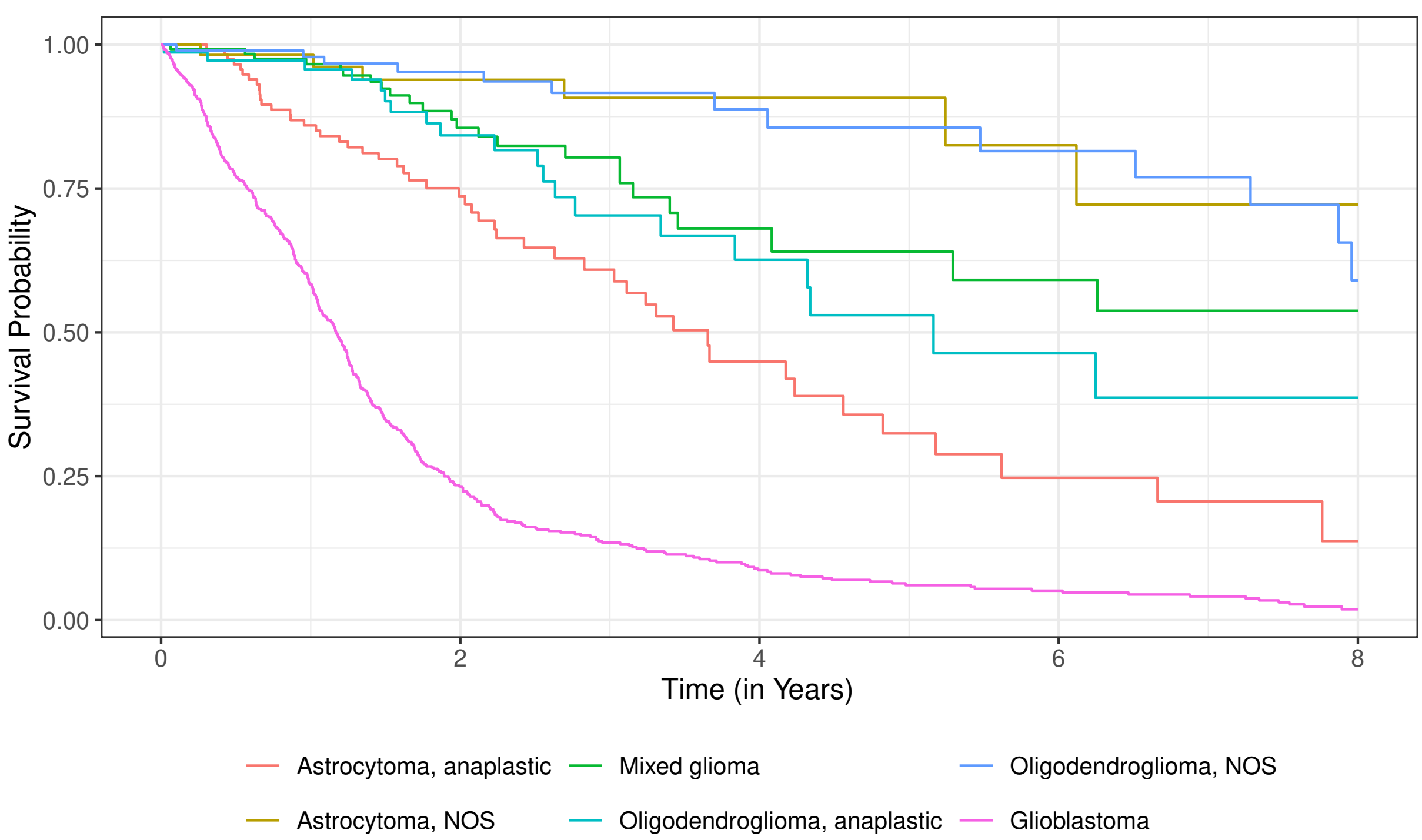

Figure S 11: Kaplan-Meier curves of the case study data set.

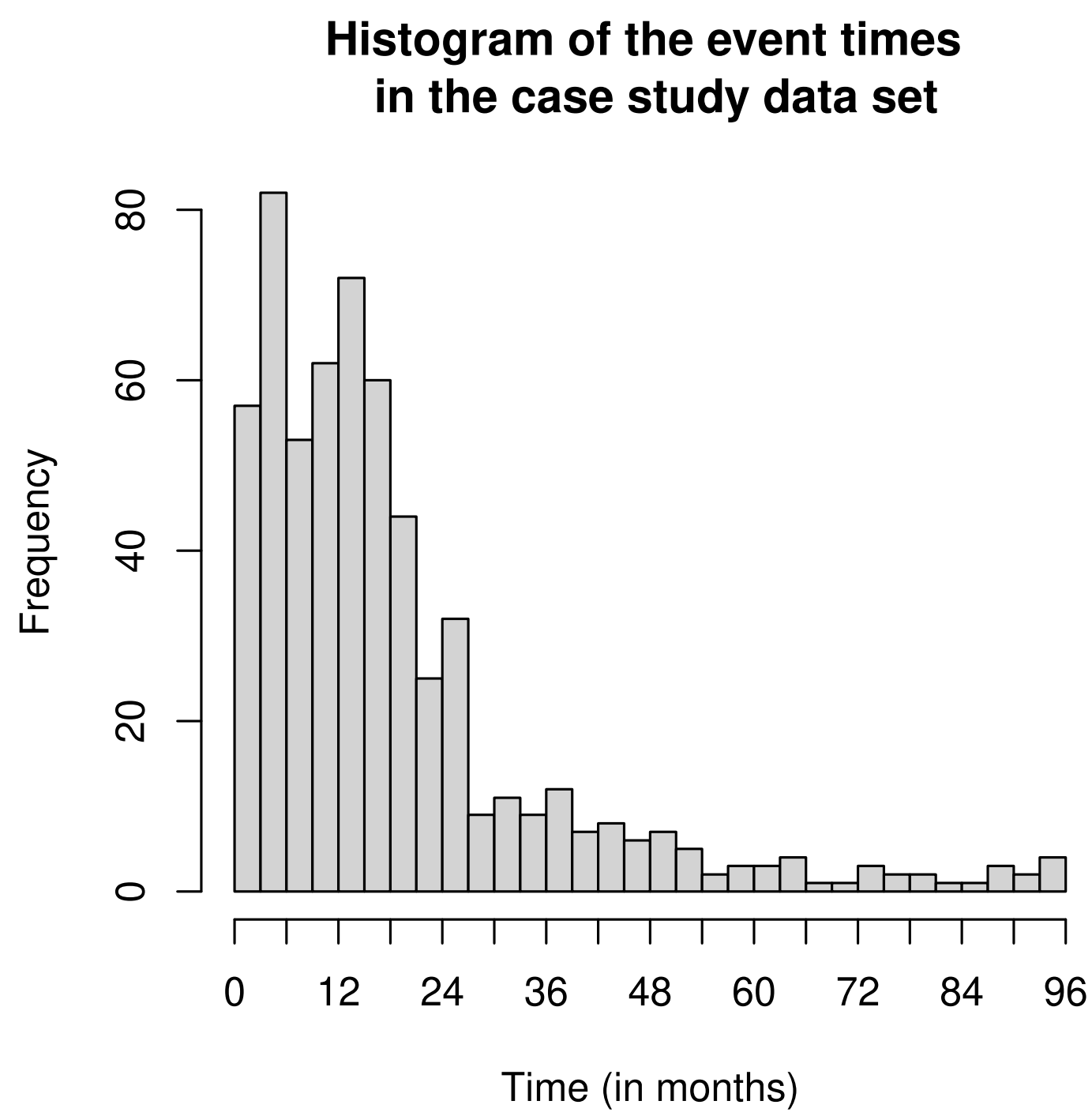

Figure S 12: Histogram of the event times in the case study data set.
