# Supplementary material for: Capturing Heterogeneous Time‐Variation in Covariate Effects in Non‐Proportional Hazard Regression Models
Source: Biom J. 2026 Sep 20;68(5):e70179. doi: 10.1002/bimj.70179 (PMC13590908; doi:10.1002/bimj.70179)
Supplement: Supplementary file 2 [file BIMJ-68-e70179-s001.zip › code_and_data/Plots_and_Tables/Figure_S8.pdf]

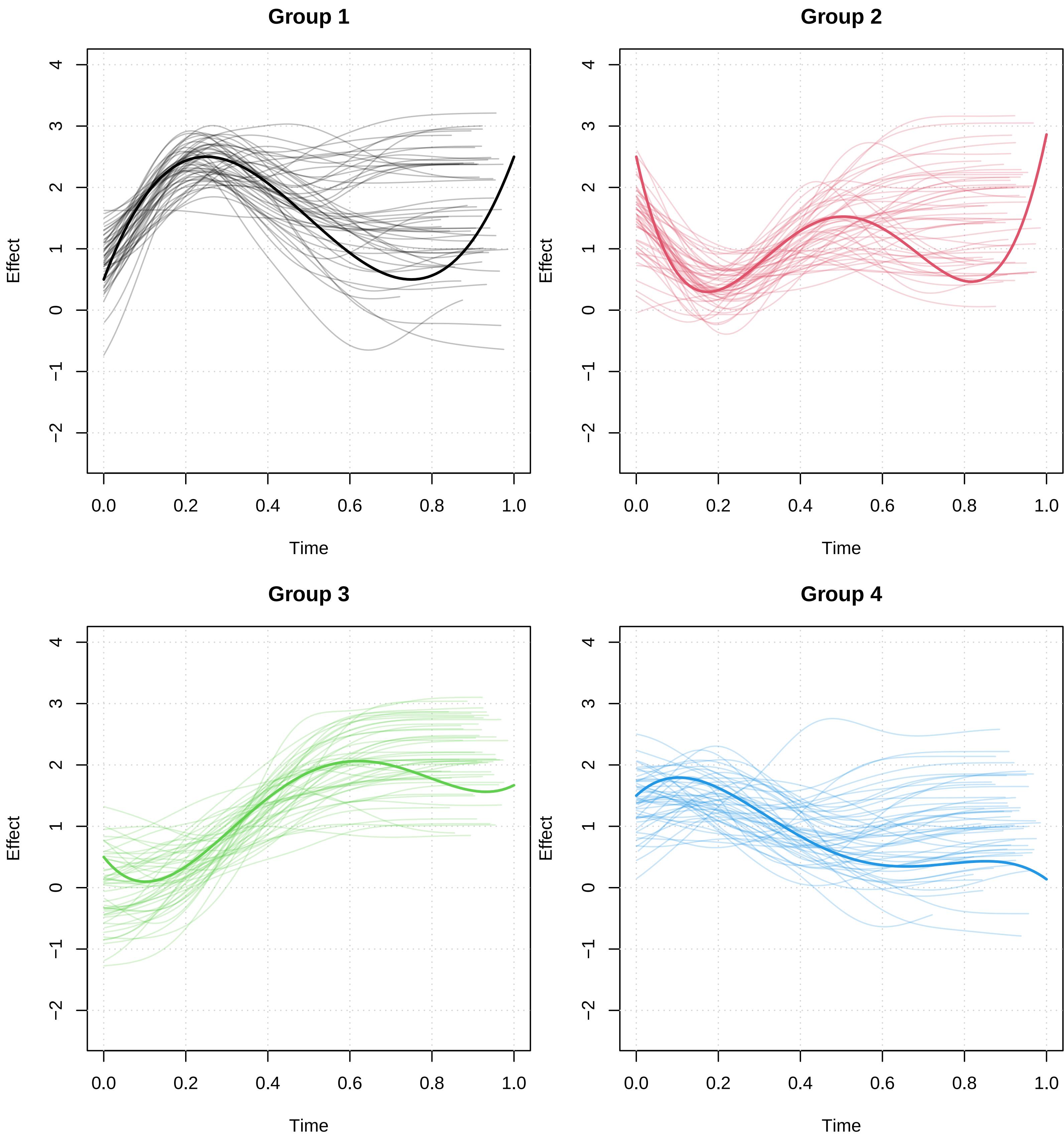
Group 1
Group 2
Group 3
Group 4
Effect
Time
4
3
2
1
0
−1
−2
0.0
0.2
0.4
0.6
0.8
1.0
