# Supplementary material for: Capturing Heterogeneous Time‐Variation in Covariate Effects in Non‐Proportional Hazard Regression Models
Source: Biom J. 2026 Sep 20;68(5):e70179. doi: 10.1002/bimj.70179 (PMC13590908; doi:10.1002/bimj.70179)
Supplement: Supplementary file 2 [file BIMJ-68-e70179-s001.zip › code_and_data/README.pdf]

README for

Capturing heterogeneous time-variation in covariate effects in non-proportional hazard regression models

by Niklas Hagemann, Thomas Kneib and Kathrin Möllenhoff

Setup

- R version
  - 4.5.1
- Packages: version:
  - dplyr 1.2.0
  - ggsurvfit 1.2.0
  - CoxFlexBoost 0.7-0
  - pammtools 0.7.4
  - gratia 0.11.2
  - foreach 1.5.2
  - pec 2025.06.24
  - DoseFinding 1.4-1
  - doParallel 1.0.17
  - ggplot2 4.0.2
  - Survival 3.8-3
  - mgcv 1.9-4
- All packages except CoxFlexBoost are available from CRAN.
  - CoxFlexBoost is available from R-Forge via: `install.packages("CoxFlexBoost", repos="http://R-Forge.R-project.org")`
- All provided scripts assume that the current working directory is set to the folder this README file is stored in.

File Structure

- The folder this readme file is stored in contains:
  - **simulation_functions.R** and **plot_functions.R**
    - Two R scripts containing functions that are sourced by the other R scripts to conduct the simulation study and produce the figures, respectively.
  - **pec_bug_fix.R**
    - One R script fixing an error that occurs when using the pec::pec method on a pammtools::pamm object and is sourced by the other R scripts.
  - **simulation.R**
    - An R script that runs all simulations.
  - **simulation.sh**
    - A Slurm batch script that can be used to run the simulations on a Linux cluster.
  - **plots_simulation.R**
    - One R script that produces all figures and tables of Section 4 including the corresponding figures in the supplementary materials.

- **case_study.R**
    - An R script that runs the whole case study including the production of all figures and tables of Section 5 including the corresponding figures in the supplementary materials.
- **DGP_plots.R**
    - An R script that produces Figures 1 and S1.
- A **folder structure** for data, results, plots and tables
    - All pots and tables are stored in the "Plots_and_Tables" folder.
    - The case study datasets are stored in the "Case_Study_Data" folder.
    - All R scripts store their results in the "Results" folder within this file structure.
    - A version of the "Results" folder containing intermediate results can be downloaded from Zenodo (see "Simulation Study" Section below for details).

Simulation Study

- The simulation study code is written to be executed on a Linux cluster by using the provided Slurm (Simple Linux Utility for Resource Management) batch script.
- All simulation results can be replicated by running the Simulation.sh Slurm batch script. Previously, the Slurm batch script must be adapted to the available computational resources and modules.
- Running simulation.sh executes the simulation.R script. This produces all results needed to subsequently create all figures, tables, and results shown in Section 4 of the manuscript as well as the corresponding figures in the supplementary materials. The results will be stored in the provided "Results" folder structure.
- If the simulation script simulation.R should be executed on a Windows or MacOS machine, lines 46 and 47 of simulation.R must be adapted accordingly. However, running all simulations on a desktop PC is not advised due to the high computational costs. Individual calls of simulation_run() and simulation_run_bio() can be performed in approx. 2 hours on a standard PC when deploying state-of-the-art hardware (e.g. a 10 core Apple M5).
- Because running all simulations has high computational costs, the intermediate results produced by simulation.R are stored on Zenodo and accessible from the DOI http://doi.org/10.5281/zenodo.22015321. After downloading the intermediate results, replace the (empty) "Results" folder with the downloaded "Results" folder.
- Subsequently, all figures and tables shown in Section 4 of the manuscript can be produced by running plot_simulation.R. The figures and tables will be stored in the Plots_and_Tables folder.

Case Study

- The data used in the case study chapter of the paper was downloaded from cBioPortal on August 14, 2024. Minor data updates have been made since then, such that the current version on cBioPortal differs slightly from the version stored in the "Case_Study_Data" folder and used in the paper.
- The current version of the data can be downloaded from https://www.cbioportal.org/study/clinicalData?id=difg_tcga_gdc and https://www.cbioportal.org/study/clinicalData?id=gbm_tcga_gdc.

- Running the R script CaseStudy.R produces all results, figures and tables included in Section 5 of the manuscript as well as the corresponding figures and tables in the supplementary materials.
